# Supplementary material for: An integrative epigenome-based strategy for unbiased functional profiling of clinical kinase inhibitors
Source: Mol Syst Biol. 2024 May 9;20(6):626–50. doi: 10.1038/s44320-024-00040-x (PMC11148061; doi:10.1038/s44320-024-00040-x)
Supplement: Supplementary file 1 — Appendix [file 44320_2024_40_MOESM1_ESM.pdf]

# Appendix

## An integrative epigenome-based strategy for unbiased functional profiling of clinical kinase inhibitors

Francesco Gualdrini *et al.*

*Corresponding authors. Email: francesco.gualdrini@ieo.it; gioacchino.natoli@ieo.it;

## Table of Contents

**Appendix Figure S1.** *Selection of the CKIs used in the study*................................ **2**

**Appendix Figure S2.** *CKIs' effects in stimulated macrophages*.............................. **3**

**Appendix Figure S3.** *Perturbation score calculation and evaluation of affected CREs upon LPS or IL-4 stimulation*............................................................................. **4**

**Appendix Figure S4.** *Clustering CKIs on the basis of the perturbation likelihood score (top) and the time/genomic overlaps of CKIs' effects for the IL-4-regulated CREs (bottom)*......... **6**

**Appendix Figure S5.** *Description of the transformed Features implemented in XGBoost models* **8**

**Appendix Figure S6.** *SHAP decision plot relative to Filgotinib and Midostaurin* .......... **10**

**Appendix Figure S7.** *Correlation between JAK inhibitors in RNA-seq data sets and the transcriptional and signaling effects of Midostaurin and Momelotinib* ......................... **12**

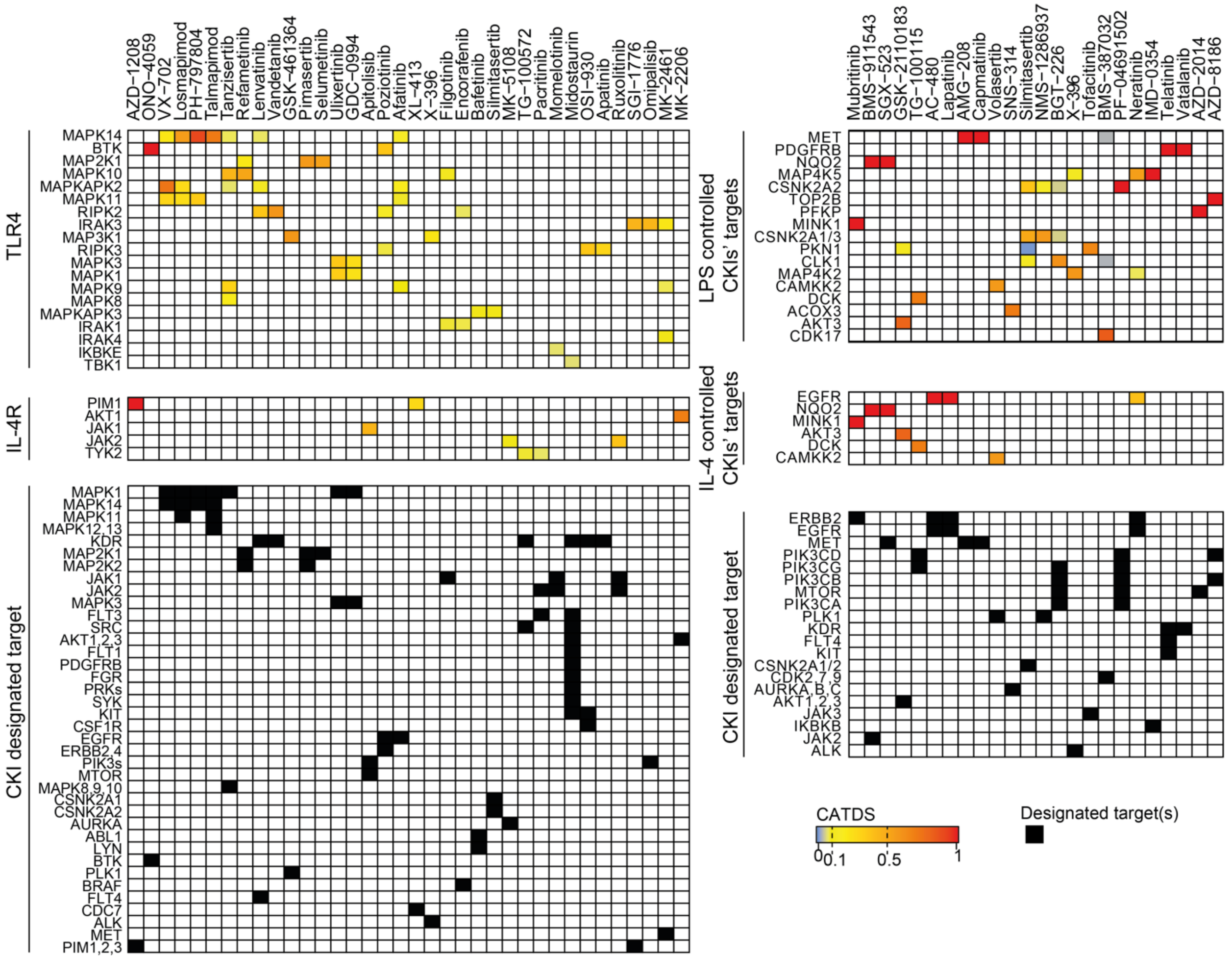


**Appendix Figure S1.** *Selection of the CKIs used in the study.*

Summary heatmaps showing the relationship between the 58 selected CKIs and TLR4 or IL-4 signaling components (left) or kinases transcriptionally regulated by either LPS or IL-4 (right). CATDS score as obtained from Klaeger et al. (Klaeger *et al*, 2017) are reported on a blue- to-orange scale. In single white/black color the designated targets per CKI are reported.

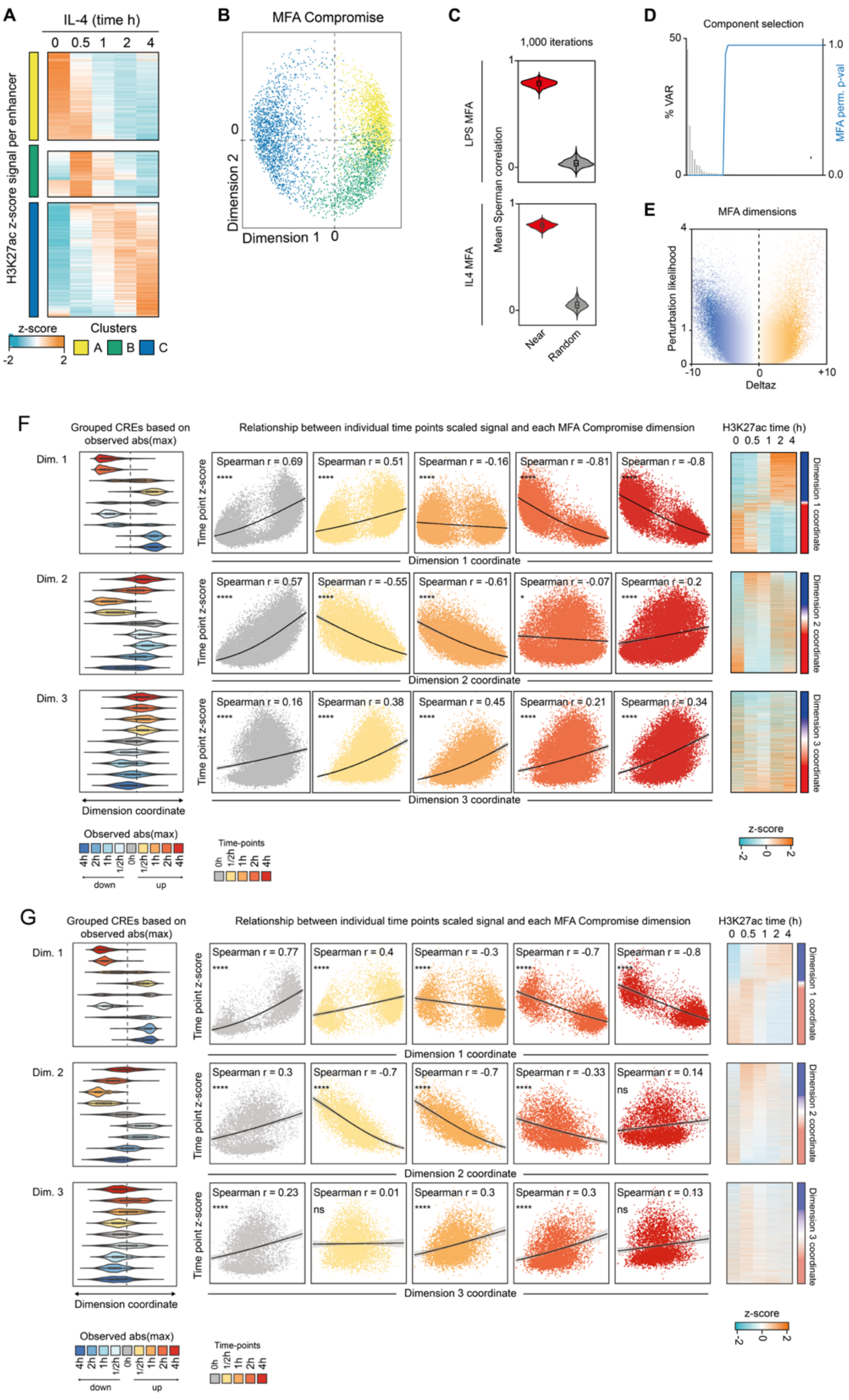


**Appendix Figure S2.** *CKIs' effects in stimulated macrophages.*

(**A**) Three main clusters of CREs with IL-4-regulated H3K27ac are shown. The three groups of CREs are color-coded.

(**B**) Reduced dimensionality plot showing the H3K27ac changes of individual CREs in macrophages stimulated with IL-4. The first two dimensions of the MFA compromised Analysis (MFA) (de Tayrac *et al*, 2009; Escofier & Pagès, 2008) for the ~5,000 IL-4 controlled CREs are represented.
(**C**) Mean spearman correlation between proximal CREs (within the MFA compromise space) and randomly sampled CREs for the LPS (top) and IL-4 (bottom) H3K27ac time course.
(**D**) MFA component selection via permutation testing (1,000 iterations). Column-wise permutation was conducted to select components carrying variance more than by random. Grey bars (left y-axis) correspond to the observed un-permuted data tables; the blue line (right y-axis) correspond to the computed probability to observe equal or more variance by randomly column-wise permuted data tables.
(**E)** Relationship between the computed permutation likelihood and the change in amplitude (computed as the delta between summed z-score for each CKI and the DMSO condition) at each CRE.
**(F)** Comparison between MFA dimension and individual LPS time points. i) Violin plots dividing CREs into their point of absolute maximum. ii) Scatter plot between MFA dimension coordinates and the scaled H3K27ac Chip-seq signal by time point (within each scatter a logistic regression was performed and we report the Spearman r and the significance of the fitting). iii) Heatmap of the z-score H3K27ac Chip-seq signal by time point of all CREs ranked by the individual MFA dimensions.
**(G)** As panel F but for the IL4 time course

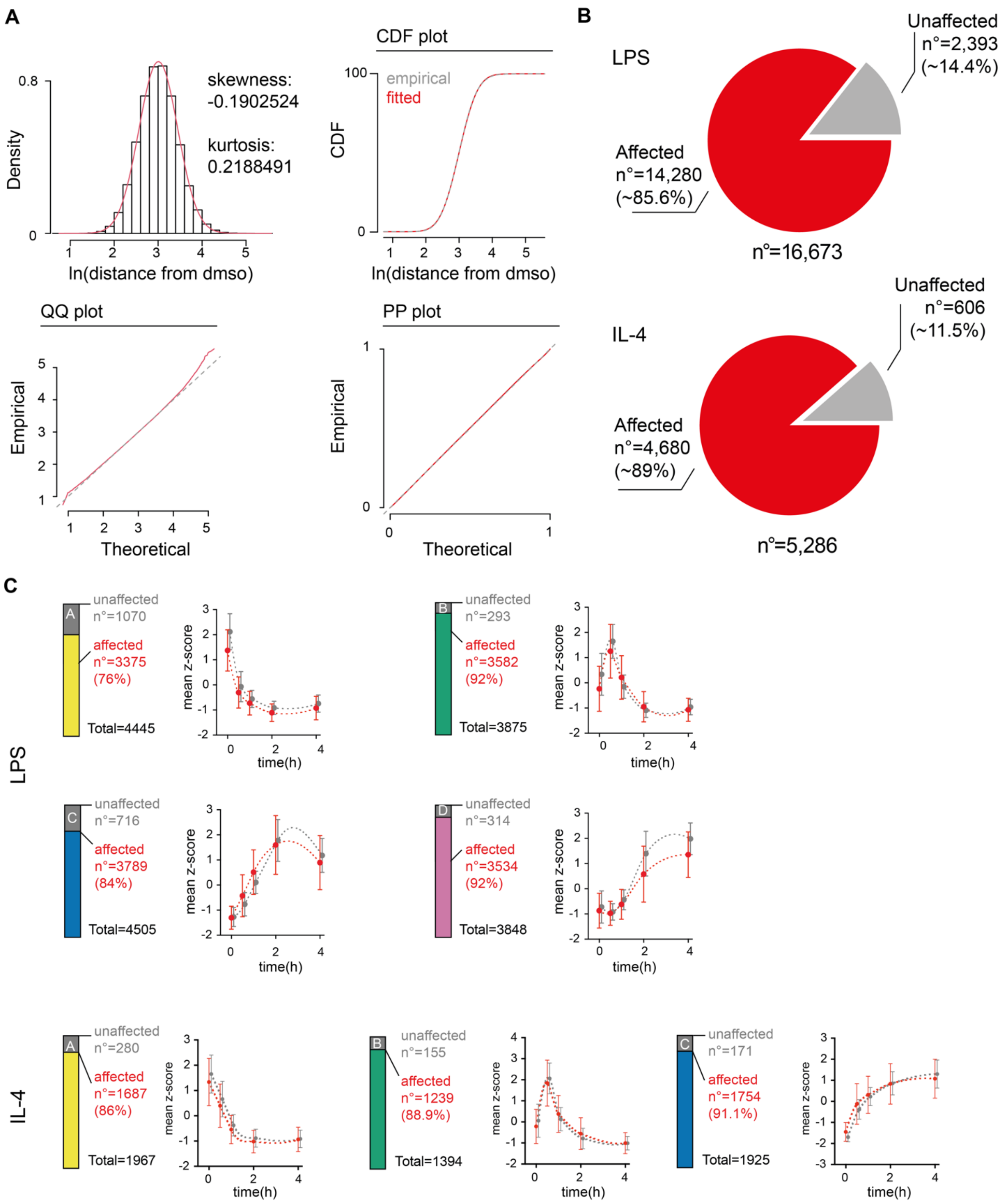


**Appendix Figure S3.** *Perturbation score calculation and evaluation of affected CREs upon LPS or IL-4 stimulation.*

(**A**) Histogram showing the frequency distribution (density) of all the computed distances to the DMSO per CRE (log-normal scale). Cumulative distribution function (CDF), quantile-quantile plot (QQ plot) and the Probability-

Probability plot, or Percent-Percent plot (PP plot) are also shown (the plots refer to the distances within the MFA compromise for the LPS conditions, similar plots were retrieved for the IL-4 conditions, data not shown).
(**B**) Pie-charts displaying the proportion of affected *vs.* unaffected CREs considering either LPS stimulation (top) or IL-4 stimulation (bottom).
(**C**) Comparison of the kinetics of affected and unaffected CREs stratified by the identified macro-clusters (4 for the LPS condition and 3 for the IL-4 conditions).

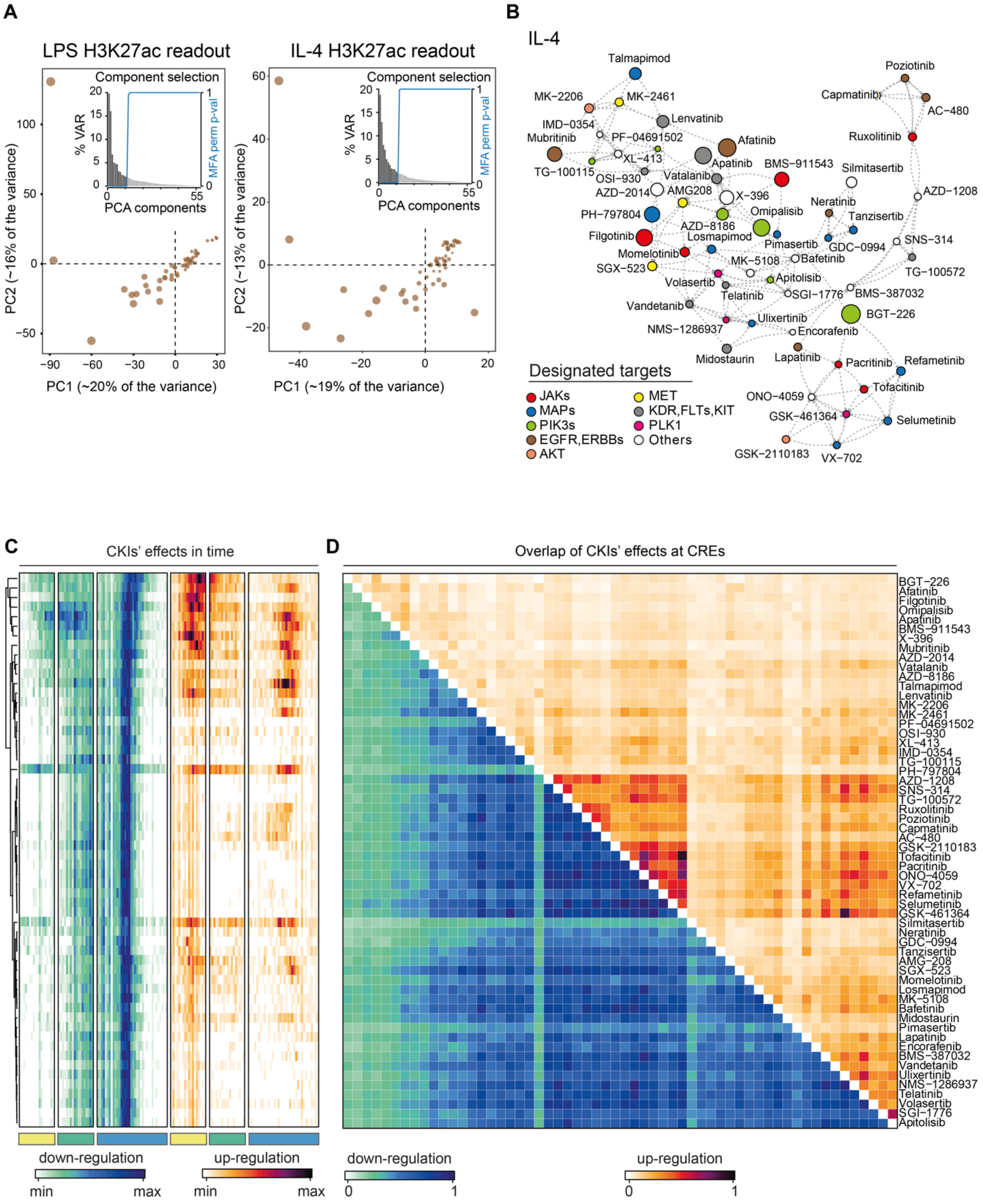


**Appendix Figure S4.** *Clustering CKIs on the basis of the perturbation likelihood score (top) and the time/genomic overlaps of CKIs' effects for the IL-4-regulated CREs (bottom).*

(**A**) Scatter plot displaying the first two components of the PCA for both the LPS (left) and IL-4 (right) conditions, conducted on the matrix reporting the perturbation likelihood per CKI. Inlet to each scatter plot is the histogram showing the selected components on the basis of iterative permutation testing.
(**B**) Two-dimensional network graph (KNN computed on the basis of the perturbation likelihood per CKI) showing the mutual relationships among CKIs. Each CKI is a vertex connected to the three closest CKIs. Vertexes are colored according to the Designated Target Kinase.
(**C)** Time-resolved effects of CKIs on H3K27ac at IL-4-regulated CREs. The effects of CKIs on H3K27ac at the four main groups of CREs (as shown in **Figure S1B-C**) are reported. CKIs were clustered based on the computed perturbation likelihood at each CREs. Cluster branching and naming are identical to panel **B**.
(**D**) Genomic overlap of CKIs' effects at IL-4-regulated CREs. CKIs were clustered based on the computed perturbation likelihood at each CREs.

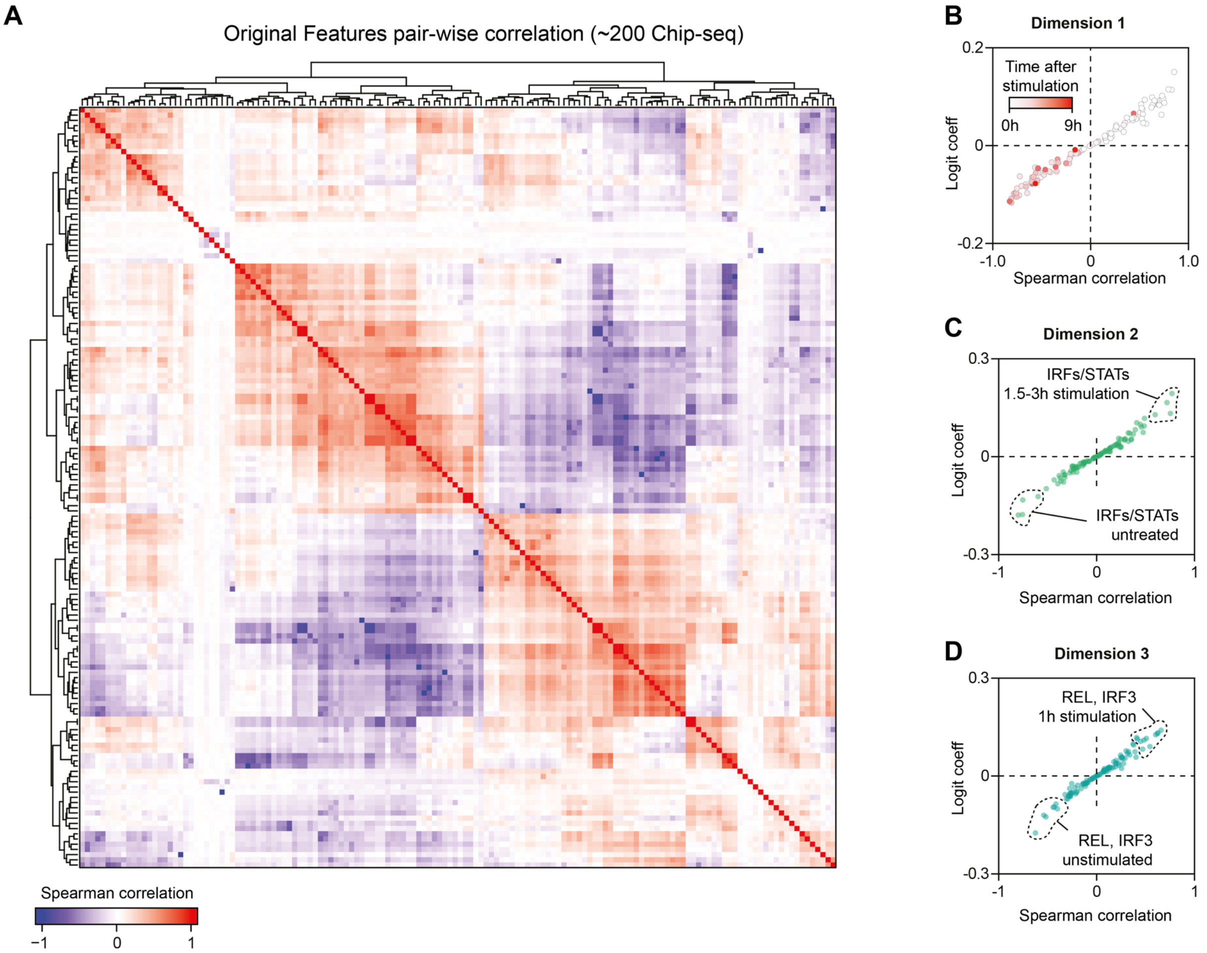


**Appendix Figure S5.** *Description of the transformed Features implemented in XGBoost models.*

(**A**) Pair-wise correlation of the un-transformed features. Several features are highly correlated with each other making multi-collinearity an issue to address, resolved with the MFA dimensionality reduction strategy.
(**B**) Description of the first feature used to train each model. Reported is the Spearman correlation (x-axes) and the logit coefficient (y-axes) between each original feature and the specific dimension of the MFA is reported with a scatter plot. Point are color coded according to the time of stimulation with the effective separation between untreated (white) and treated TFs Chip-seq samples.
(**C**) Description of the second feature used to train each model. Reported is the Spearman correlation (x-axes) and the logit coefficient (y-axes) between each original feature and the specific dimension of the MFA is reported with a scatter plot. IRFs/STATs stimulated Chip-seq samples are effectively separated from untreated samples.
(**D**) Description of the third feature used to train each model. Reported is the Spearman correlation (x-axes) and the logit coefficient (y-axes) between each original feature and the specific dimension of the MFA is reported with a scatter plot. IRF3/REL stimulated Chip-seq samples are effectively separated from untreated samples.

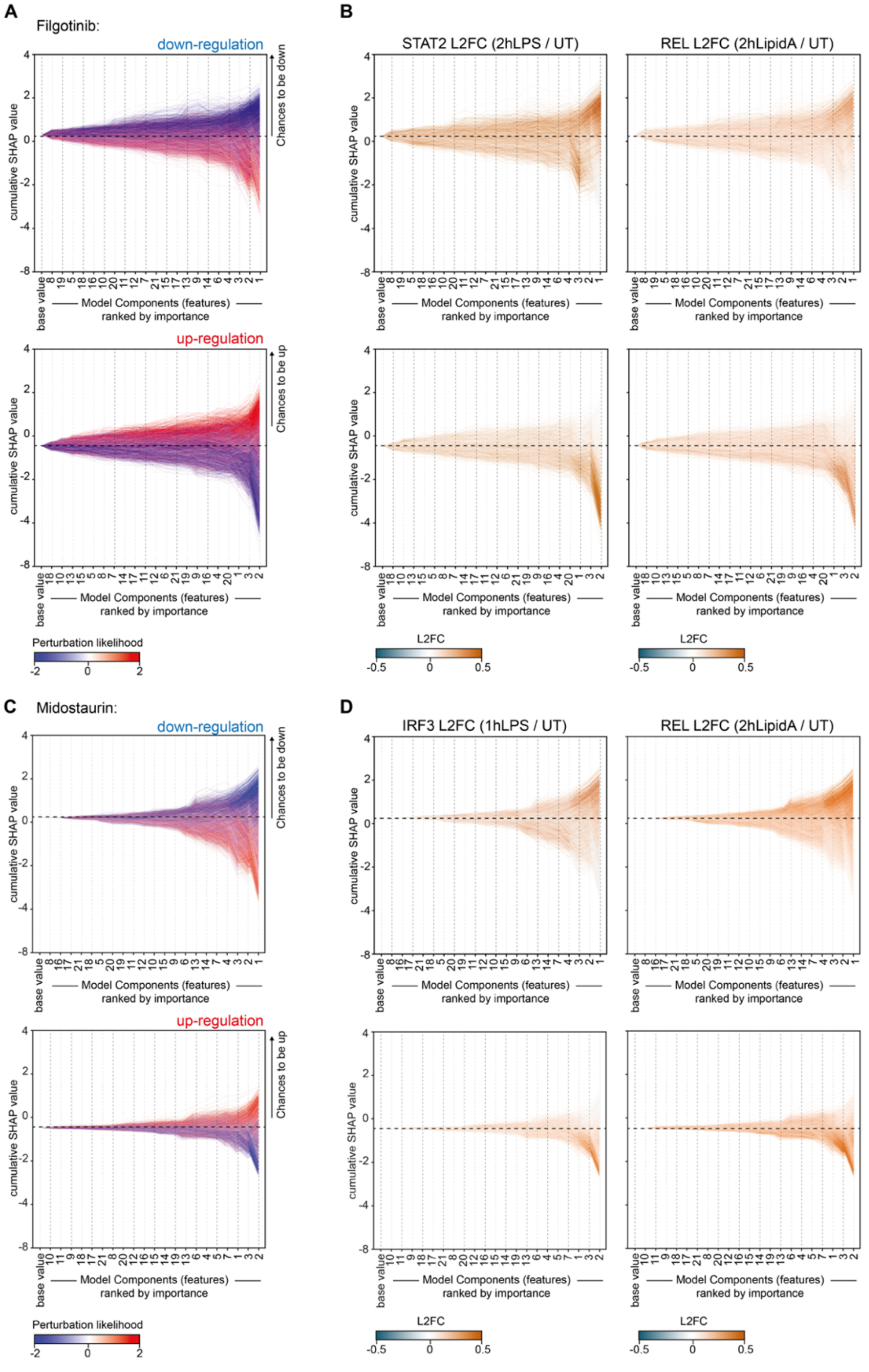


**Appendix Figure S6**. *SHAP decision plot relative to Filgotinib and Midostaurin*.
(**A**) Trajectory of each CRE considering the cumulative SHAP values as a function of the features (ranked by importance). Each CRE is colored according to the perturbation score associated with Filgotinib treatment. The top panel reports the SHAPley values associated with the prediction of down-regulatory category while the bottom panel reports the SHAPley values associated with the prediction of up-regulatory category.

(**B**) Trajectory for the individual CREs as in panel A coloured by the Log2FoldChange of STAT2 induced binding (left) or REL induced binding (right) upon either LPS or LipidA stimulation.
(**C**) Trajectory of each CRE considering the cumulative SHAP values as a function of the features (ranked by importance). Each CRE is colored according to the perturbation score associated with Filgotinib treatment. The top panel reports the SHAPley values associated with the prediction of down-regulatory category while the bottom panel reports the SHAPley values associated with the prediction of up-regulatory category.
(**D**) Trajectory for the individual CREs as in panel A coloured by the Log2FoldChange of IRF3 induced binding (left) or REL induced binding (right) upon either LPS or LipidA stimulation.

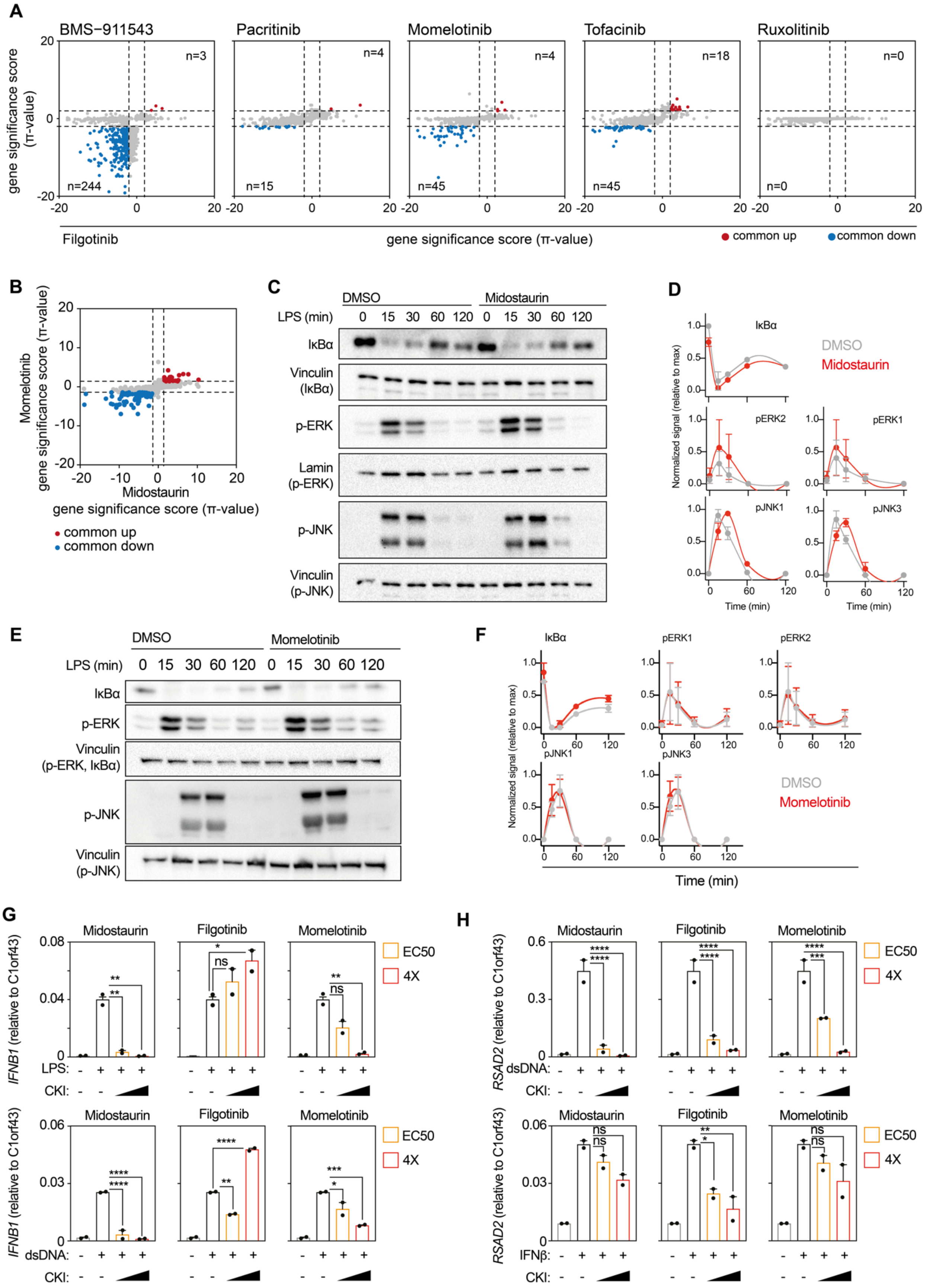
A
BMS−911543
Pacritinib
Momelotinib
Tofacinib
Ruxolitinib
gene significance score (π-value)
Filgotinib
common up
common down
B
Momelotinib gene significance score (π-value)
Midostaurin gene significance score (π-value)
C
DMSO
Midostaurin
LPS (min)
IκBα
Vinculin (IκBα)
p-ERK
Lamin (p-ERK)
p-JNK
Vinculin (p-JNK)
D
Normalized signal (relative to max)
Time (min)
E
DMSO
Momelotinib
Vinculin (p-ERK, IκBα)
F
pERK1
pERK2
pJNK1
pJNK3
G
IFNB1 (relative to C1orf43)
Midostaurin
Filgotinib
Momelotinib
EC50
4X
LPS:
dsDNA:
CKI:
H
RSAD2 (relative to C1orf43)
IFNβ:

**Appendix Figure S7**. *Correlation between JAK inhibitors in RNA-seq data sets and the transcriptional and signaling effects of Midostaurin and Momelotinib.*
(**A**) Scatter plots displaying the relationship between gene significance scores (π-value as the RNA-seq -$Log_{10}$(adjpval)*sign($Log_2$FC)) for the JAKi Filgotinib (x-axis) and each JAKi (y-axis). Genes down-regulated by both inhibitors for each pair are shown in blue while those up-regulated are shown in red (adjpval≤0.05).
(**B**) Scatter plots displaying the relationship between the gene significance scores (π-value as the RNA-seq -$Log_{10}$(adjpval)*sign($Log_2$FC)) for the Midostaurin (x-axis) and Momelotinib (y-axis). Genes down-regulated by both inhibitors are shown in blue while those up-regulated are shown in red (adjpval≤0.05).
(**C-D**) Signal transduction pathways in DMSO- and Midostaurin-treated macrophages following LPS stimulation at the indicated time points. Whole cell lysates analyzed by western blot with the indicated antibodies. Panel C shows the quantification of western blots from two independent replicates.
(**E-F**) As in panel B-C but in cells treated with either vector (DMSO) or Momelotinib.
**(G)** *IFNB1* gene expression determined by RT-qPCR following LPS (top) or dsDNA (bottom) treatment in the presence of Midostaurin, Filgotinib or Momelotinib, each at concentrations corresponding to the EC50 (orange) or 4xEC50 (red) for the intended target. Bars display the mean±SEM of two independent replicates. Significance was evaluated with Dunnett's multiple comparisons test: 0.03 (*), 0.002 (**), 0.0002 (***), p<0.0001 (****). Data are expressed relative to the expression of the housekeeping gene *C1orf43*.
**(H)** *RSAD2* gene expression determined by RT-qPCR following dsDNA (top) or IFNb (bottom) treatment in the presence of Midostaurin, Filgotinib or Momelotinib each at the concentrations corresponding to the EC50 (orange) or 4xEC50 (red) for the intended target. Bars display the mean±SEM of two independent replicates. Significance was evaluated with Dunnett's multiple comparisons test: 0.03 (*), 0.002 (**), 0.0002 (***), p<0.0001 (****). Data are expressed relative to the expression of the housekeeping gene *C1orf43*.

## References

de Tayrac M, Le S, Aubry M, Mosser J, Husson F (2009) Simultaneous analysis of distinct Omics data sets with integration of biological knowledge: Multiple Factor Analysis approach. *BMC Genomics* 10: 32
Escofier B, Pagès J (2008) *Analyses factorielles simples et multiples. Objectifs méthodes et interprétation.* Dunod
Klaeger S, Heinzlmeir S, Wilhelm M, Polzer H, Vick B, Koenig PA, Reinecke M, Ruprecht B, Petzoldt S, Meng C *et al* (2017) The target landscape of clinical kinase drugs. *Science* 358
