# Supplementary material for: An integrative epigenome-based strategy for unbiased functional profiling of clinical kinase inhibitors
Source: Mol Syst Biol. 2024 May 9;20(6):626–50. doi: 10.1038/s44320-024-00040-x (PMC11148061; doi:10.1038/s44320-024-00040-x)
Supplement: Supplementary file 7 — Source data Fig. 6 [file 44320_2024_40_MOESM7_ESM.zip › Figure 6_Source Data/I/README.rtf]

Westernblots related to Figure 6HFor each membrane colorimetric and western chemiluminescent signal are reported in individual .tiff files.MW are highlighted on the colorimetric images while samples (temperatures) and the cropped area is highlighted for the chemiluminescent images.Two independent replicate experiment of the CETSA assays are reported and Replica 2 is the one displayed in Figure 6H.Quantification using imageLab 6.0.1 by BIO-RAD is reported in the excel file “QUANTIFICATION_CETSA_6I.xlsx”
