# Supplementary figures and images for: An integrative epigenome-based strategy for unbiased functional profiling of clinical kinase inhibitors
Source: Mol Syst Biol. 2024 May 9;20(6):626–50. doi: 10.1038/s44320-024-00040-x (PMC11148061; doi:10.1038/s44320-024-00040-x)

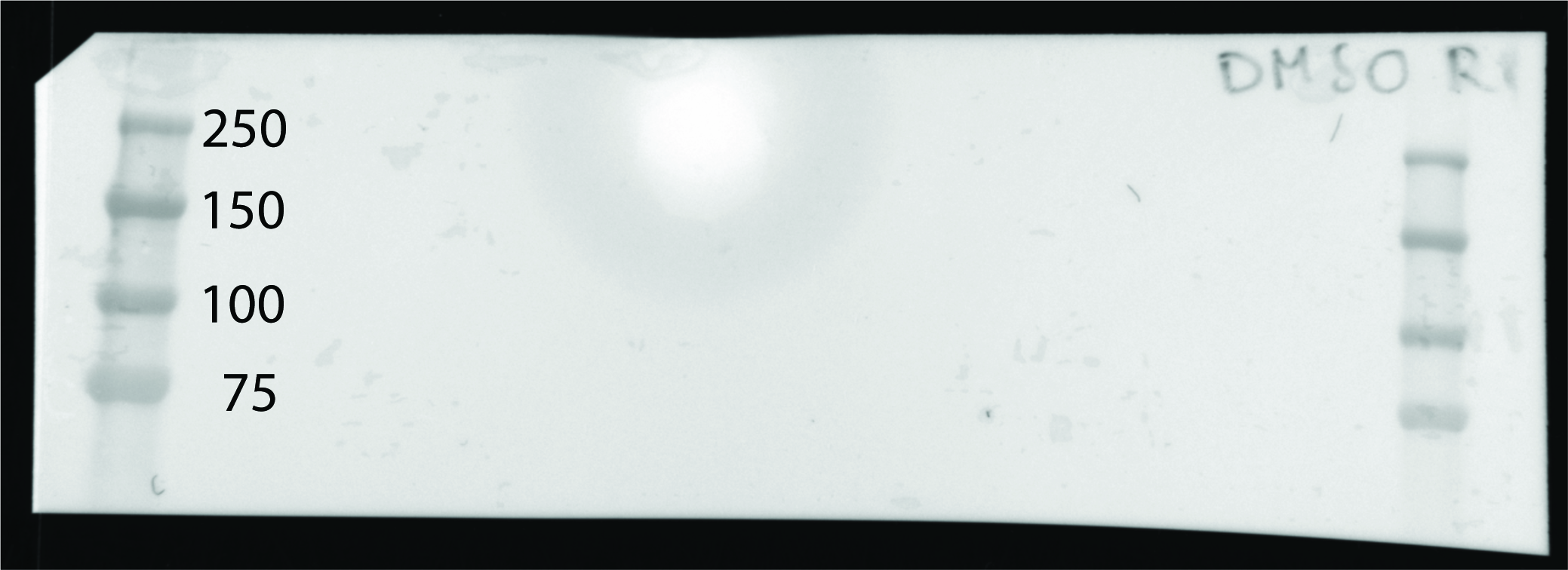

Supplement: Supplementary file 7 — Source data Fig. 6 [file 44320_2024_40_MOESM7_ESM.zip › Figure 6_Source Data/I/Image Data/colorimetric_TBK1_dmso_r1.tif]

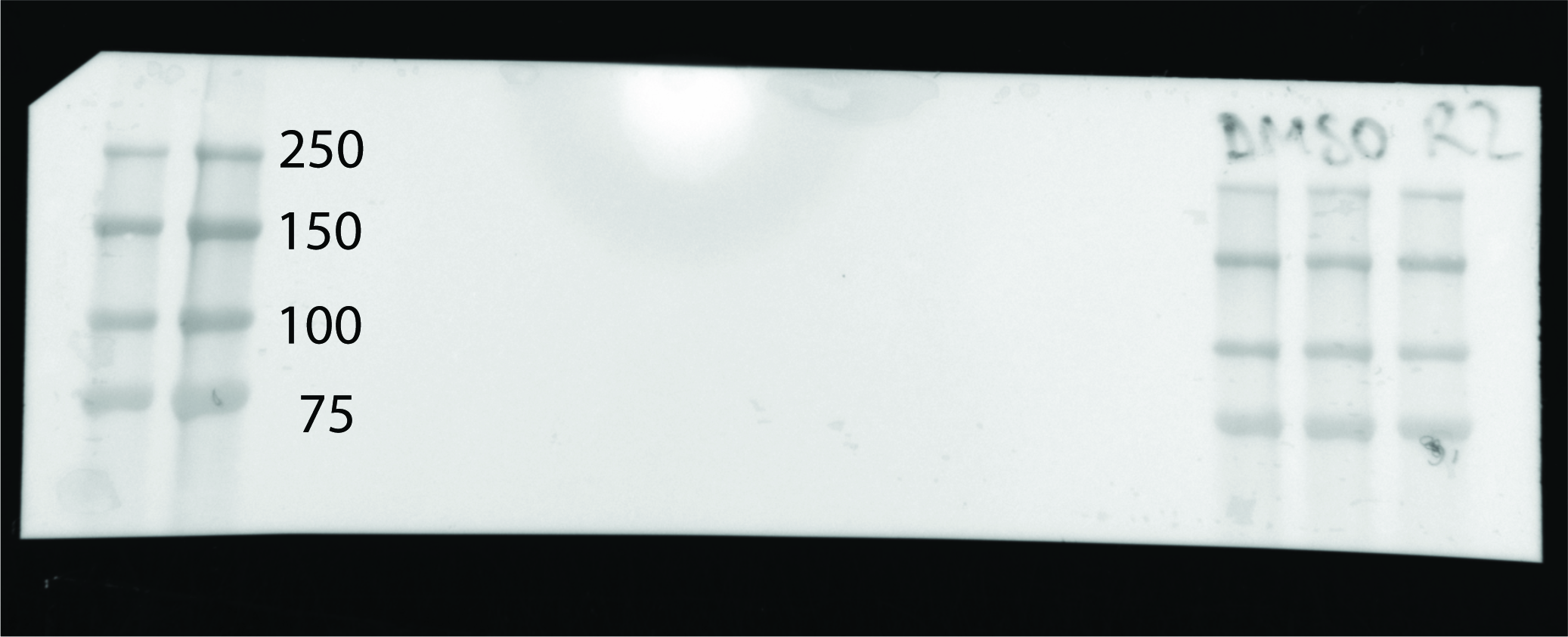

Supplement: Supplementary file 7 — Source data Fig. 6 [file 44320_2024_40_MOESM7_ESM.zip › Figure 6_Source Data/I/Image Data/colorimetric_TBK1_dmso_r2.tif]

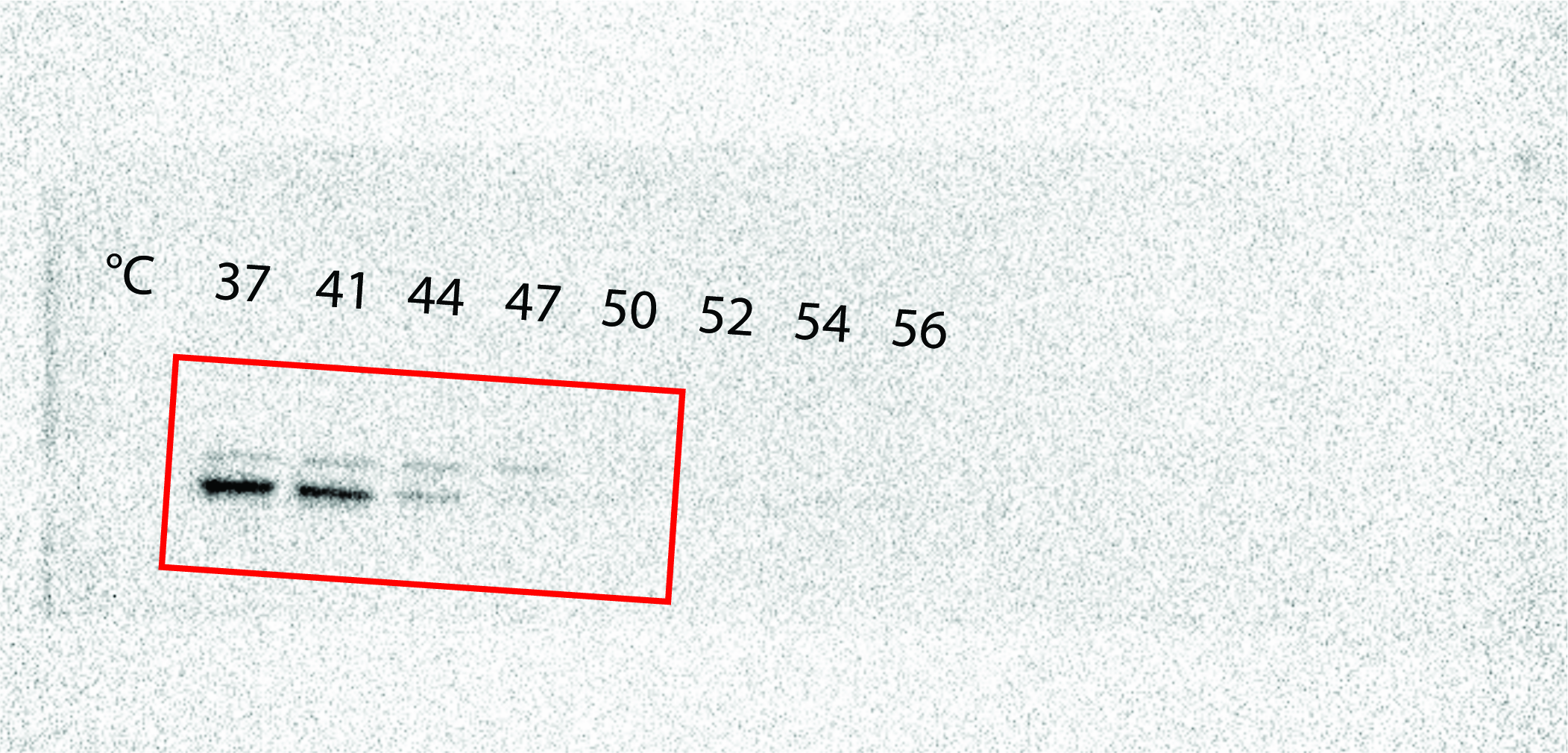

Supplement: Supplementary file 7 — Source data Fig. 6 [file 44320_2024_40_MOESM7_ESM.zip › Figure 6_Source Data/I/Image Data/western_TBK1_dmso_r1.tif]

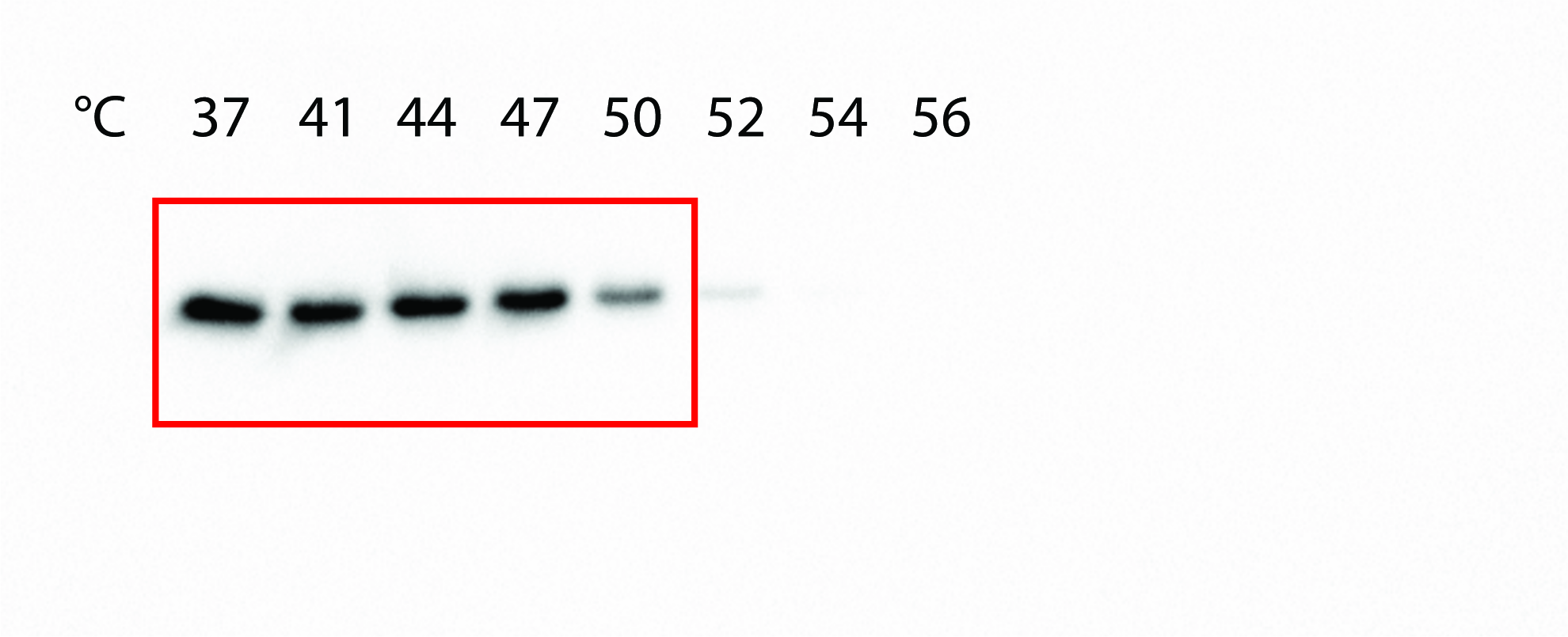

Supplement: Supplementary file 7 — Source data Fig. 6 [file 44320_2024_40_MOESM7_ESM.zip › Figure 6_Source Data/I/Image Data/western_GAPDH_dmso_r2.tif]

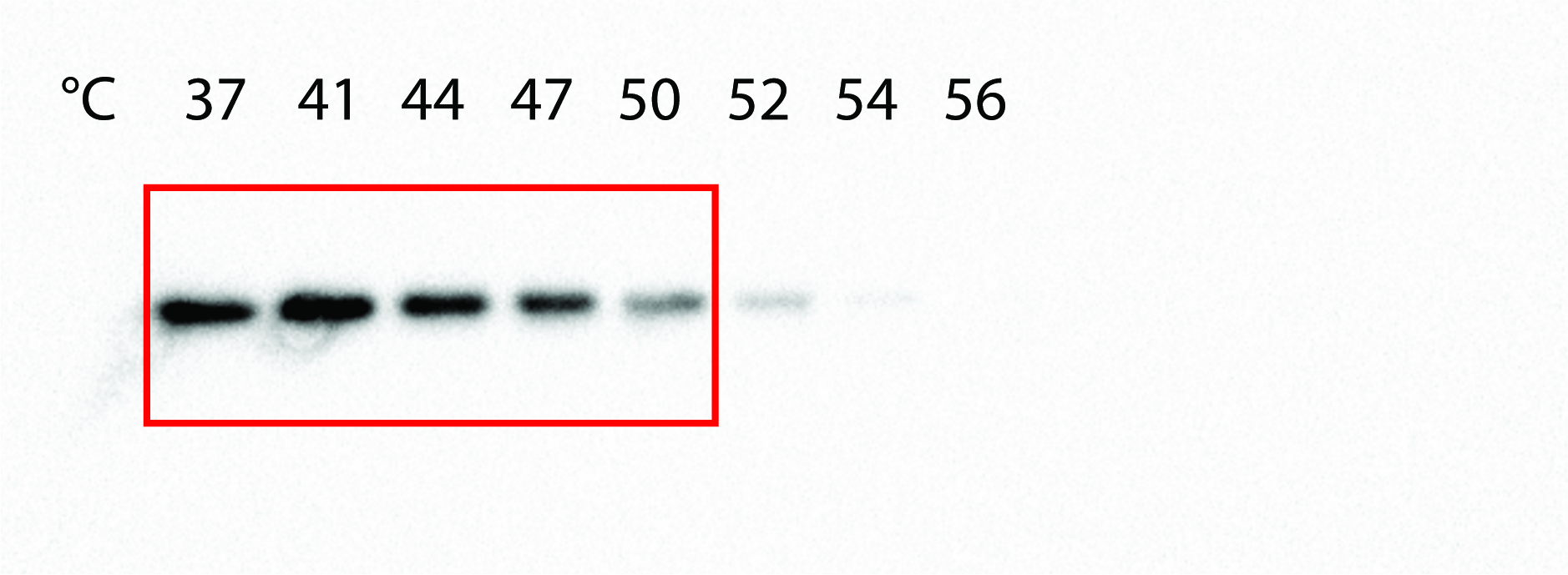

Supplement: Supplementary file 7 — Source data Fig. 6 [file 44320_2024_40_MOESM7_ESM.zip › Figure 6_Source Data/I/Image Data/western_GAPDH_momelotinib_10X_r1.tif]

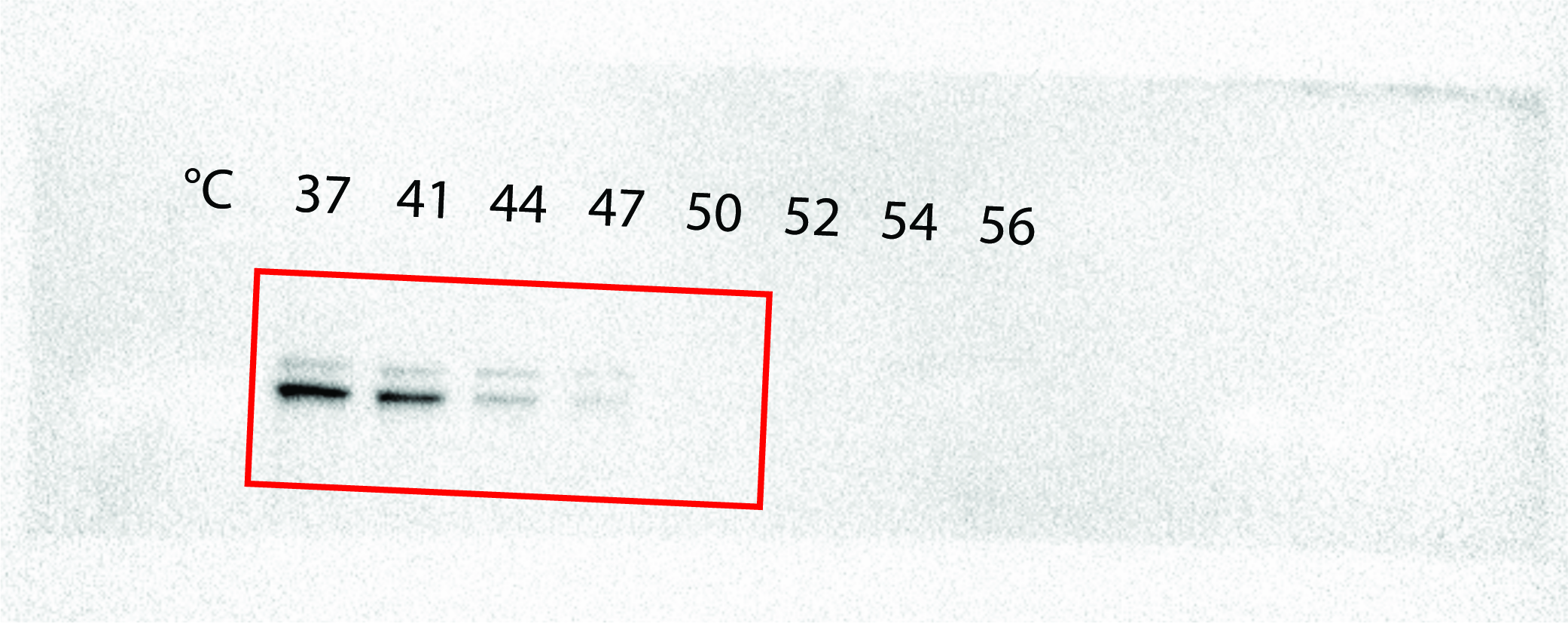

Supplement: Supplementary file 7 — Source data Fig. 6 [file 44320_2024_40_MOESM7_ESM.zip › Figure 6_Source Data/I/Image Data/western_TBK1_dmso_r2.tif]

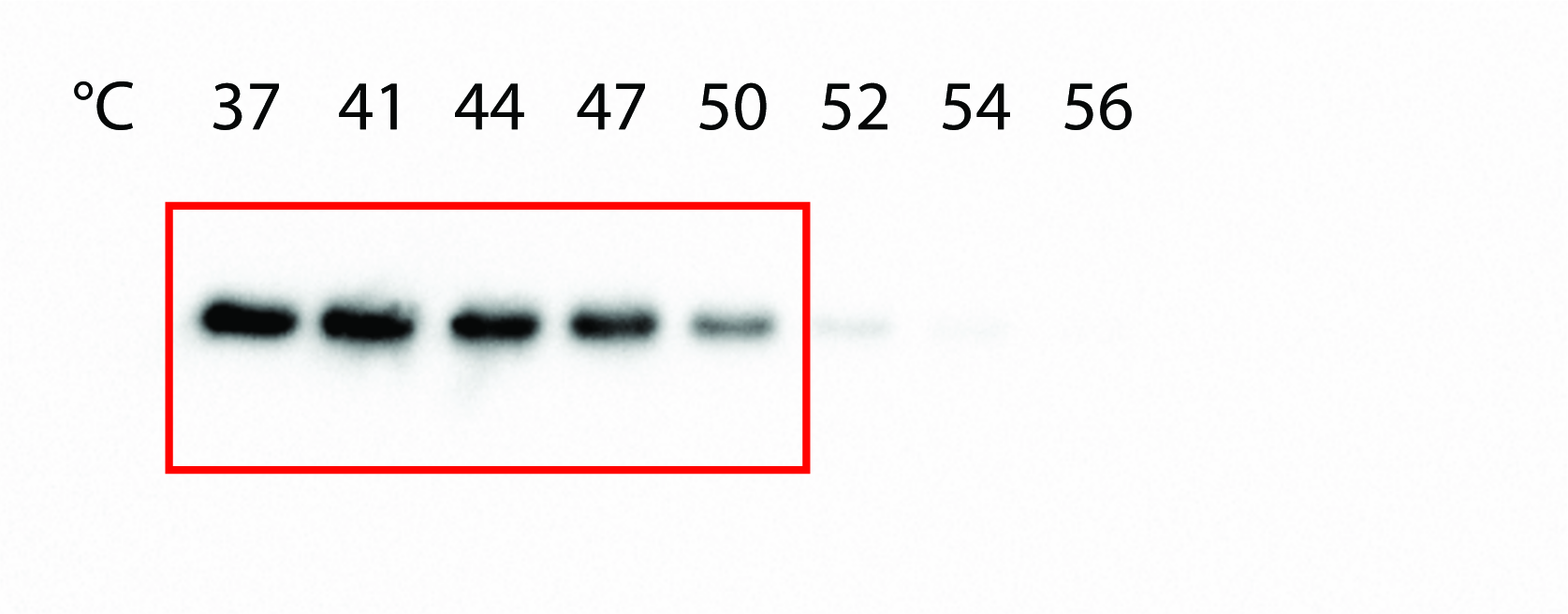

Supplement: Supplementary file 7 — Source data Fig. 6 [file 44320_2024_40_MOESM7_ESM.zip › Figure 6_Source Data/I/Image Data/western_GAPDH_dmso_r1.tif]

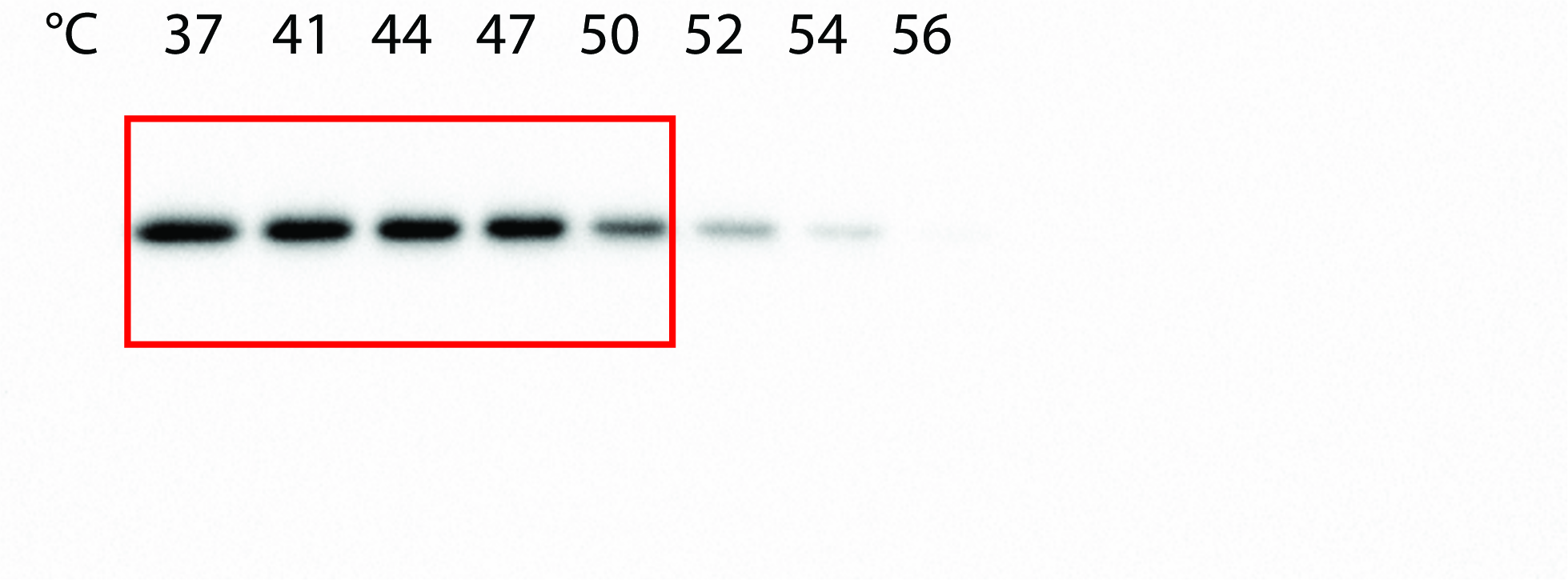

Supplement: Supplementary file 7 — Source data Fig. 6 [file 44320_2024_40_MOESM7_ESM.zip › Figure 6_Source Data/I/Image Data/western_GAPDH_momelotinib_10X_r2.tif]

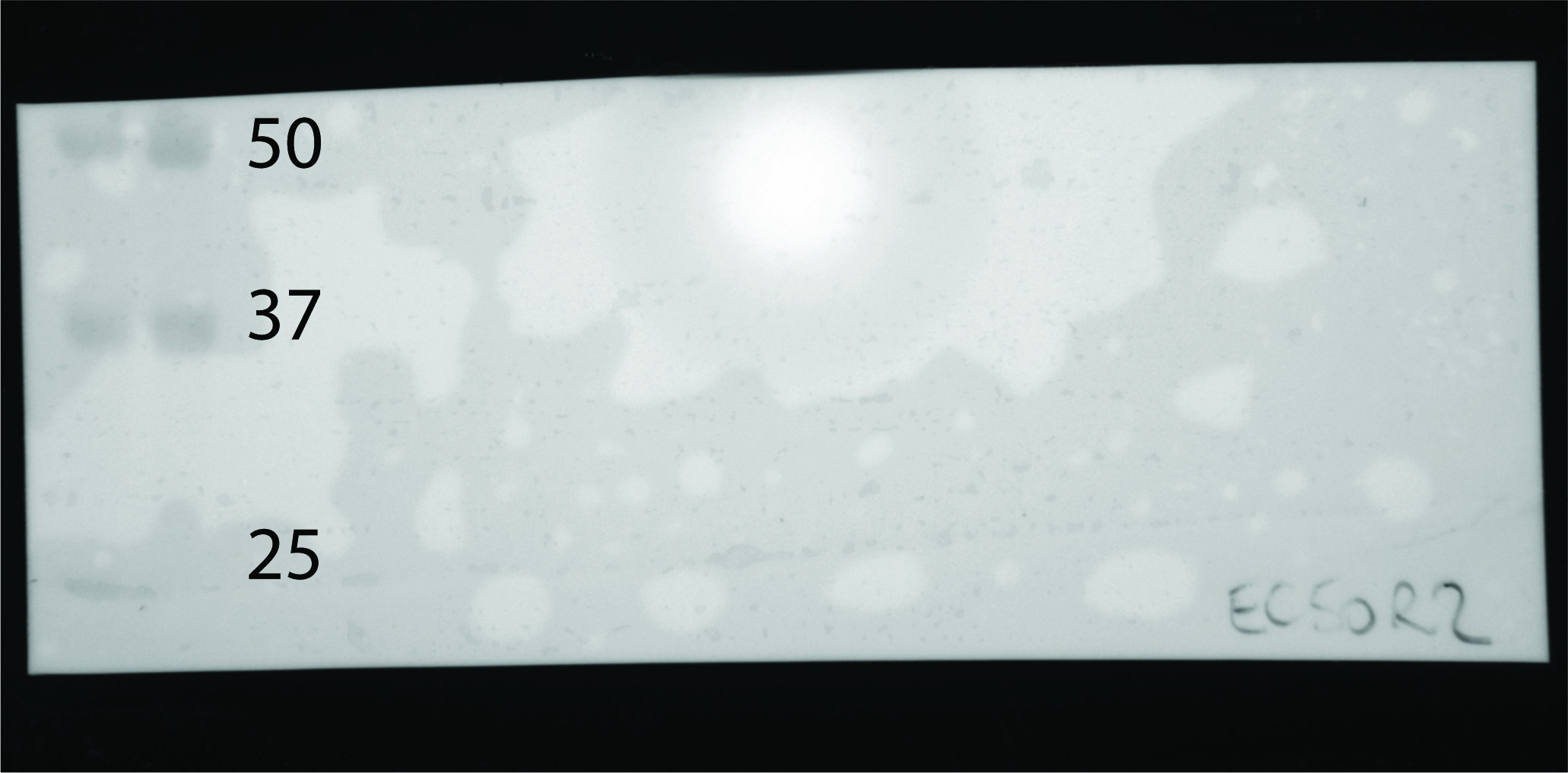

Supplement: Supplementary file 7 — Source data Fig. 6 [file 44320_2024_40_MOESM7_ESM.zip › Figure 6_Source Data/I/Image Data/colorimetric_GAPDH_momelotinib_1X_r2.tif]

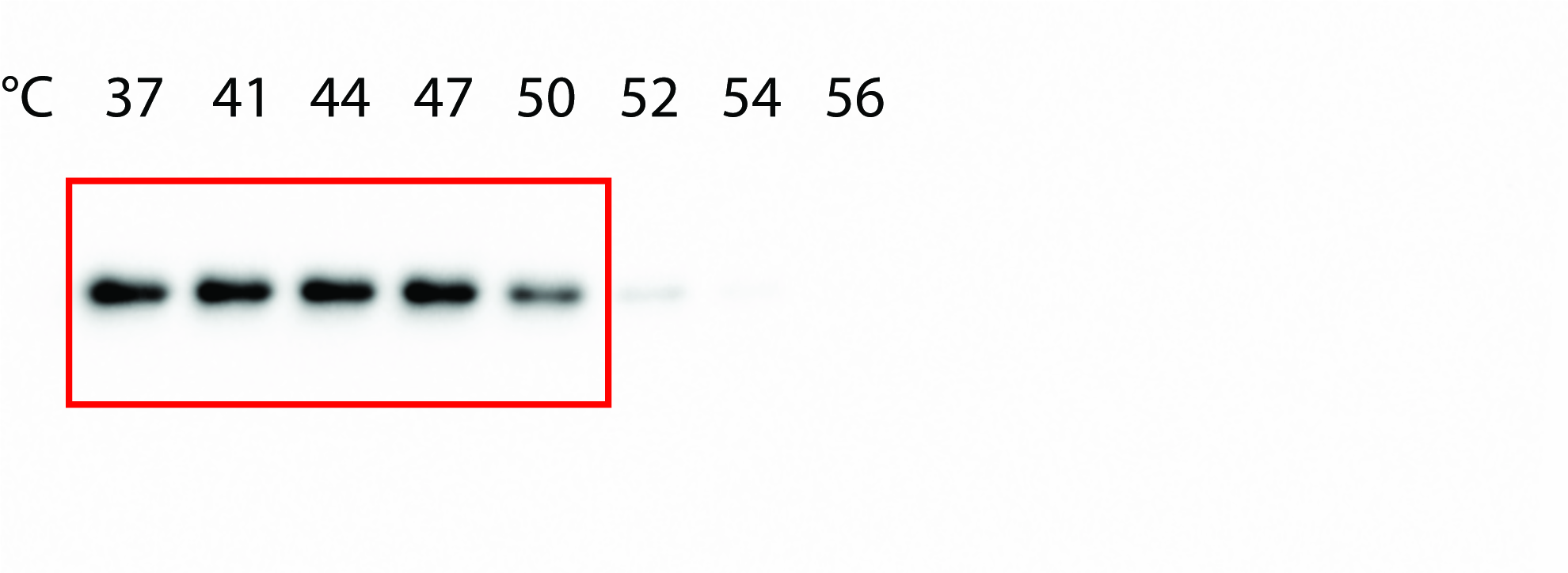

Supplement: Supplementary file 7 — Source data Fig. 6 [file 44320_2024_40_MOESM7_ESM.zip › Figure 6_Source Data/I/Image Data/western_GAPDH_momelotinib_1X_r2.tif]

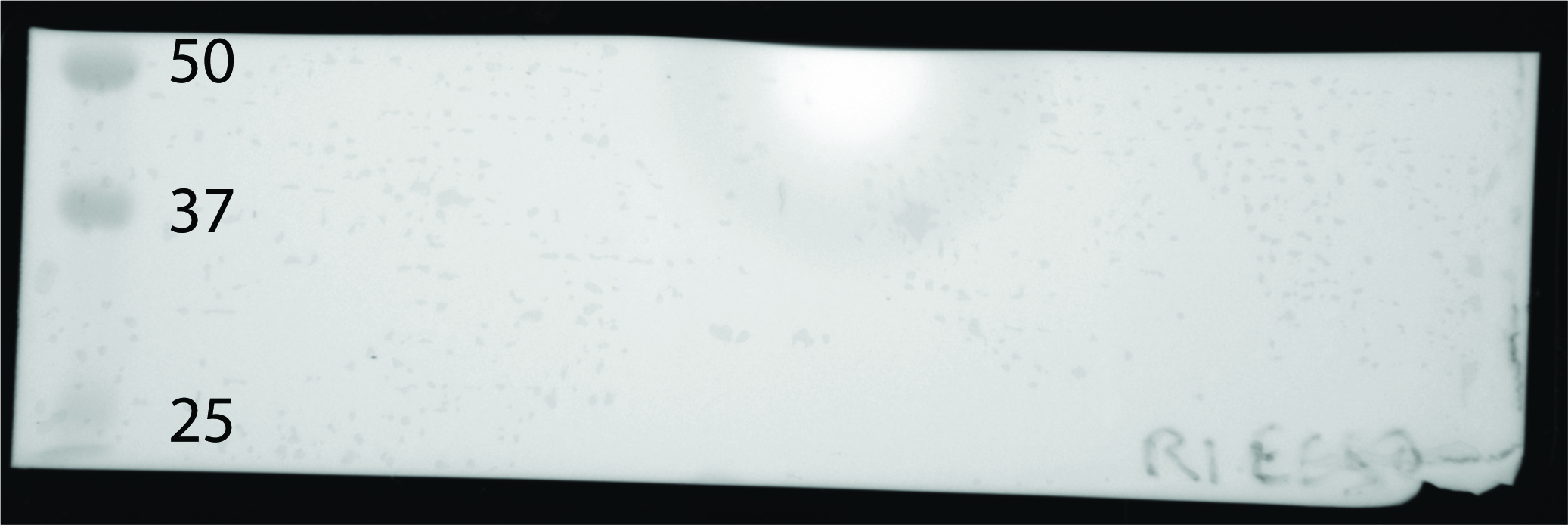

Supplement: Supplementary file 7 — Source data Fig. 6 [file 44320_2024_40_MOESM7_ESM.zip › Figure 6_Source Data/I/Image Data/colorimetric_GAPDH_momelotinib_1X_r1.tif]

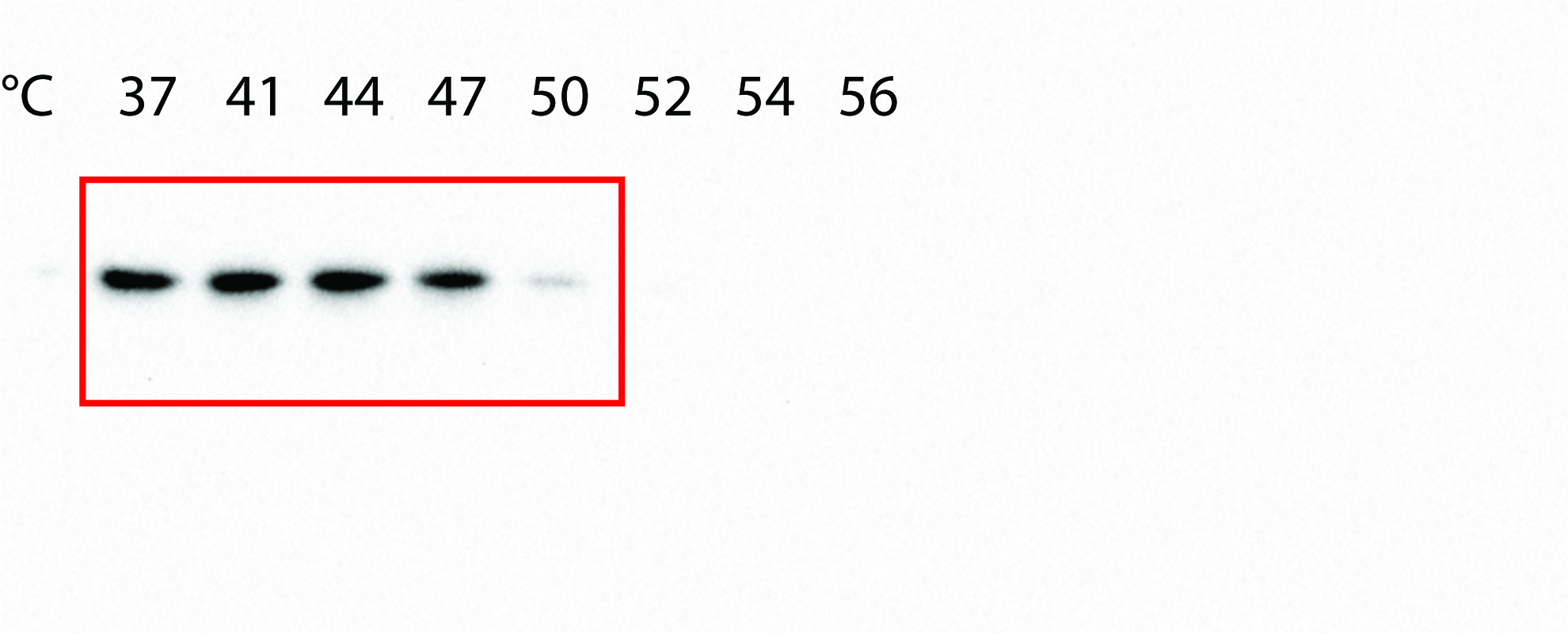

Supplement: Supplementary file 7 — Source data Fig. 6 [file 44320_2024_40_MOESM7_ESM.zip › Figure 6_Source Data/I/Image Data/western_GAPDH_momelotinib_1X_r1.tif]

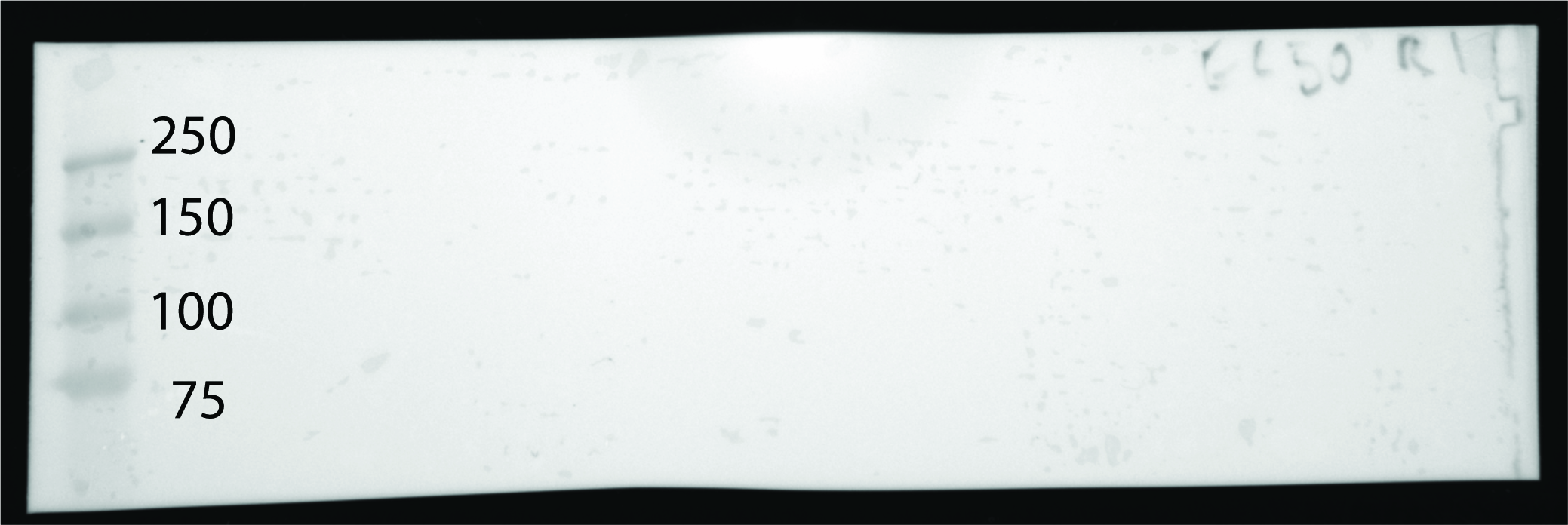

Supplement: Supplementary file 7 — Source data Fig. 6 [file 44320_2024_40_MOESM7_ESM.zip › Figure 6_Source Data/I/Image Data/colorimetric_TBK1_momelotinib_1X_r1.tif]

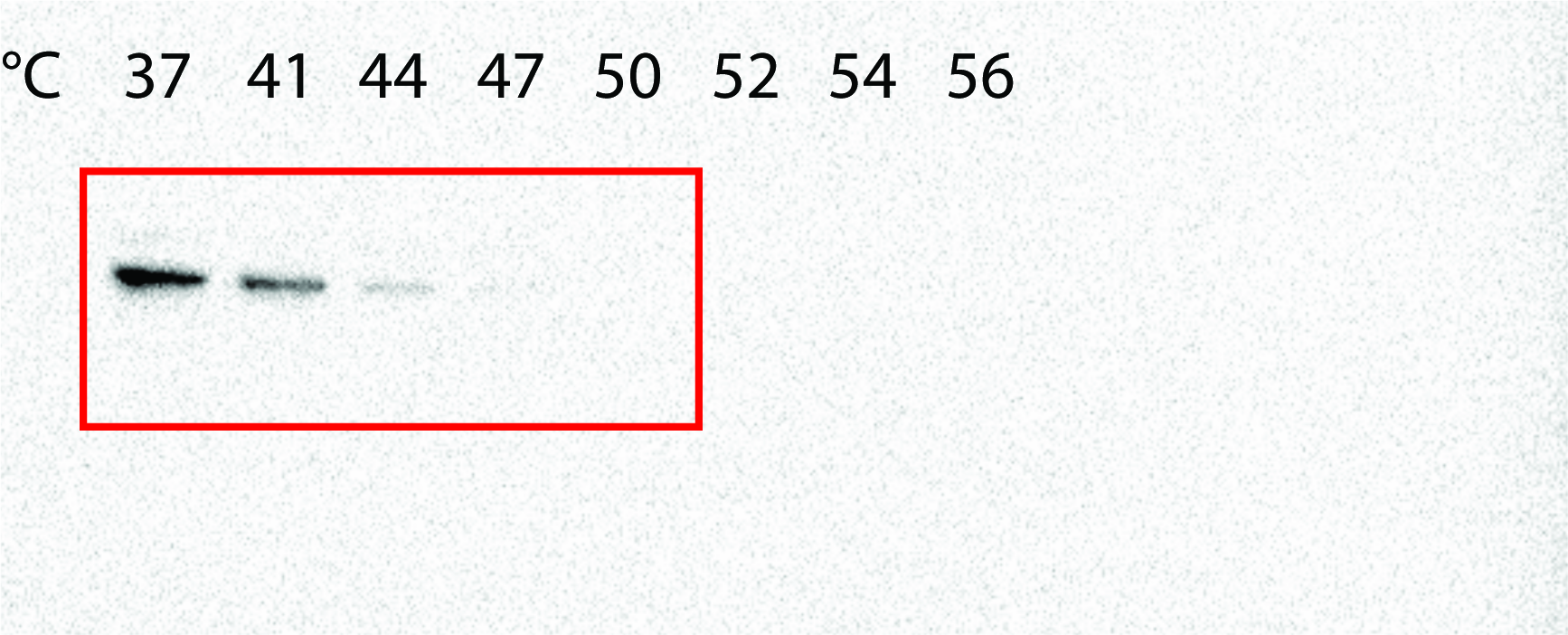

Supplement: Supplementary file 7 — Source data Fig. 6 [file 44320_2024_40_MOESM7_ESM.zip › Figure 6_Source Data/I/Image Data/western_TBK1_momelotinib_4X_r2.tif]

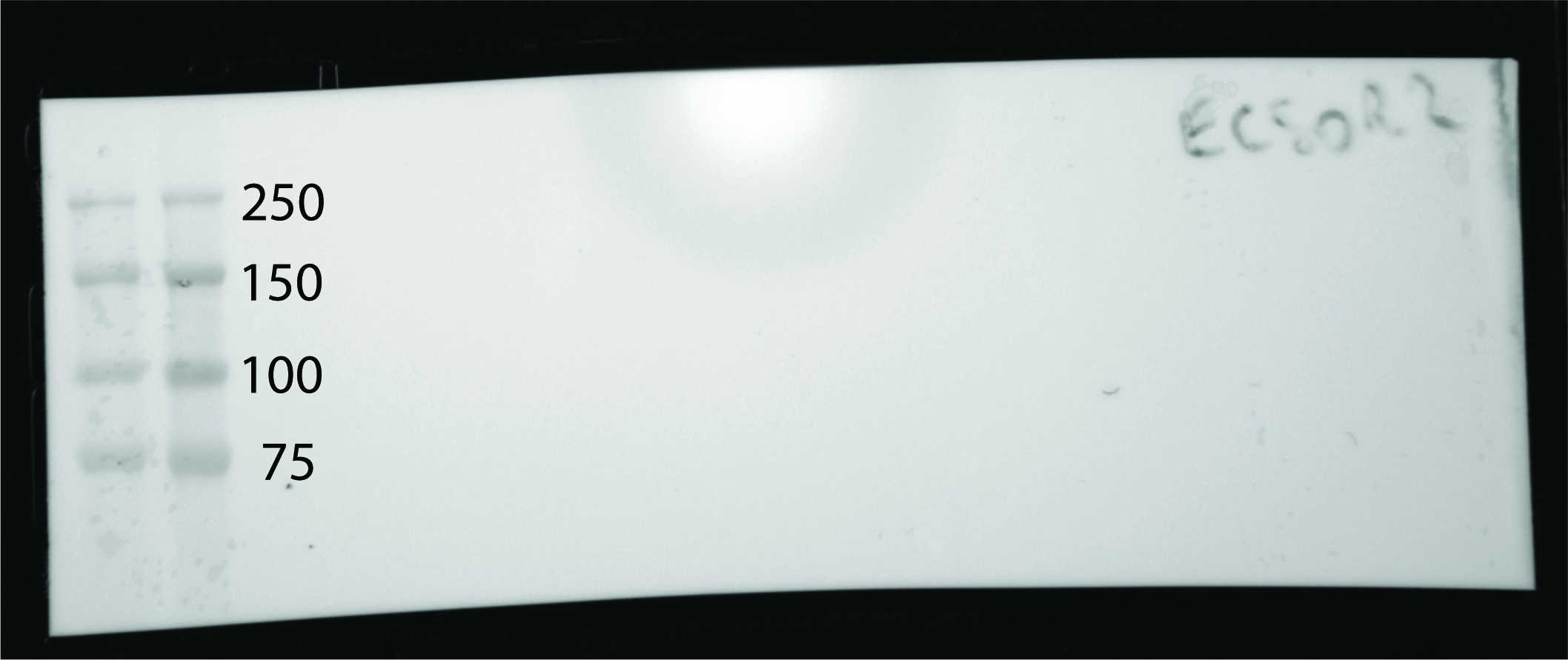

Supplement: Supplementary file 7 — Source data Fig. 6 [file 44320_2024_40_MOESM7_ESM.zip › Figure 6_Source Data/I/Image Data/colorimetric_TBK1_momelotinib_1X_r2.tif]

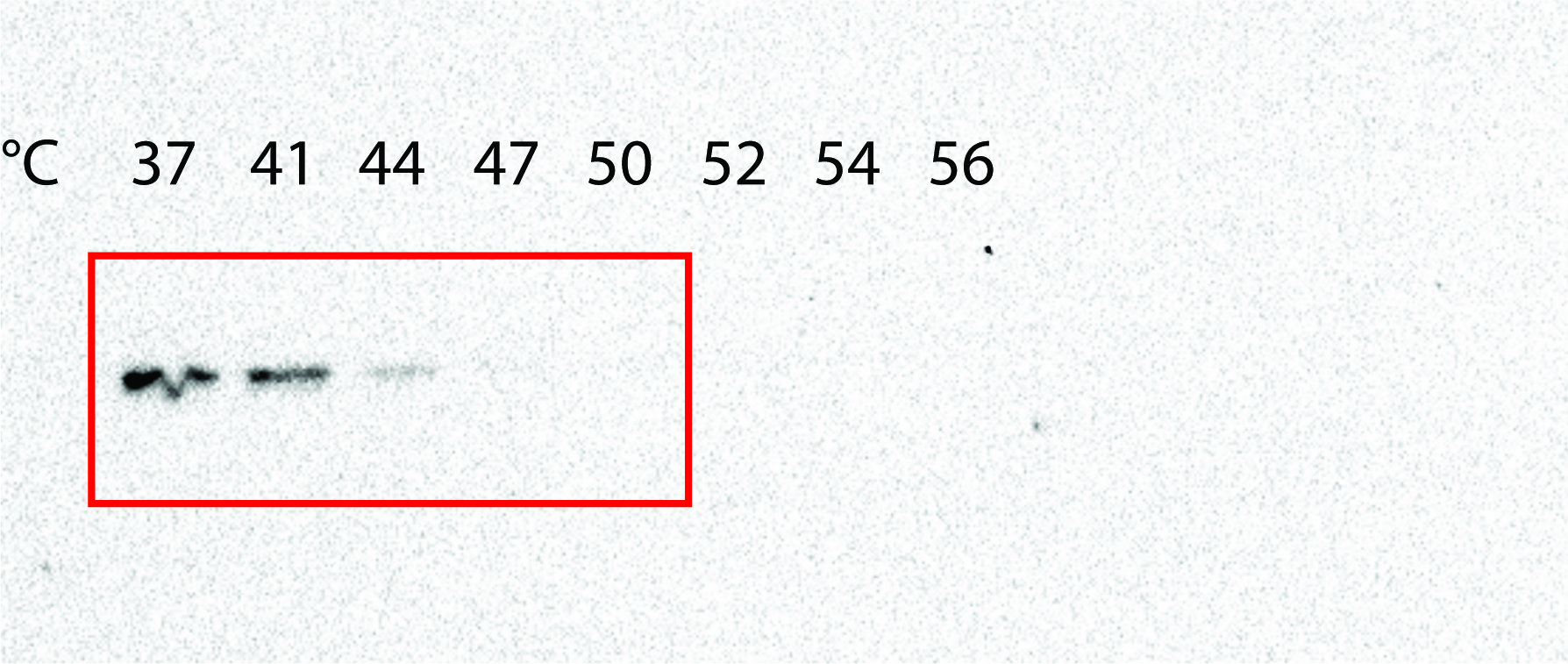

Supplement: Supplementary file 7 — Source data Fig. 6 [file 44320_2024_40_MOESM7_ESM.zip › Figure 6_Source Data/I/Image Data/western_TBK1_momelotinib_4X_r1.tif]

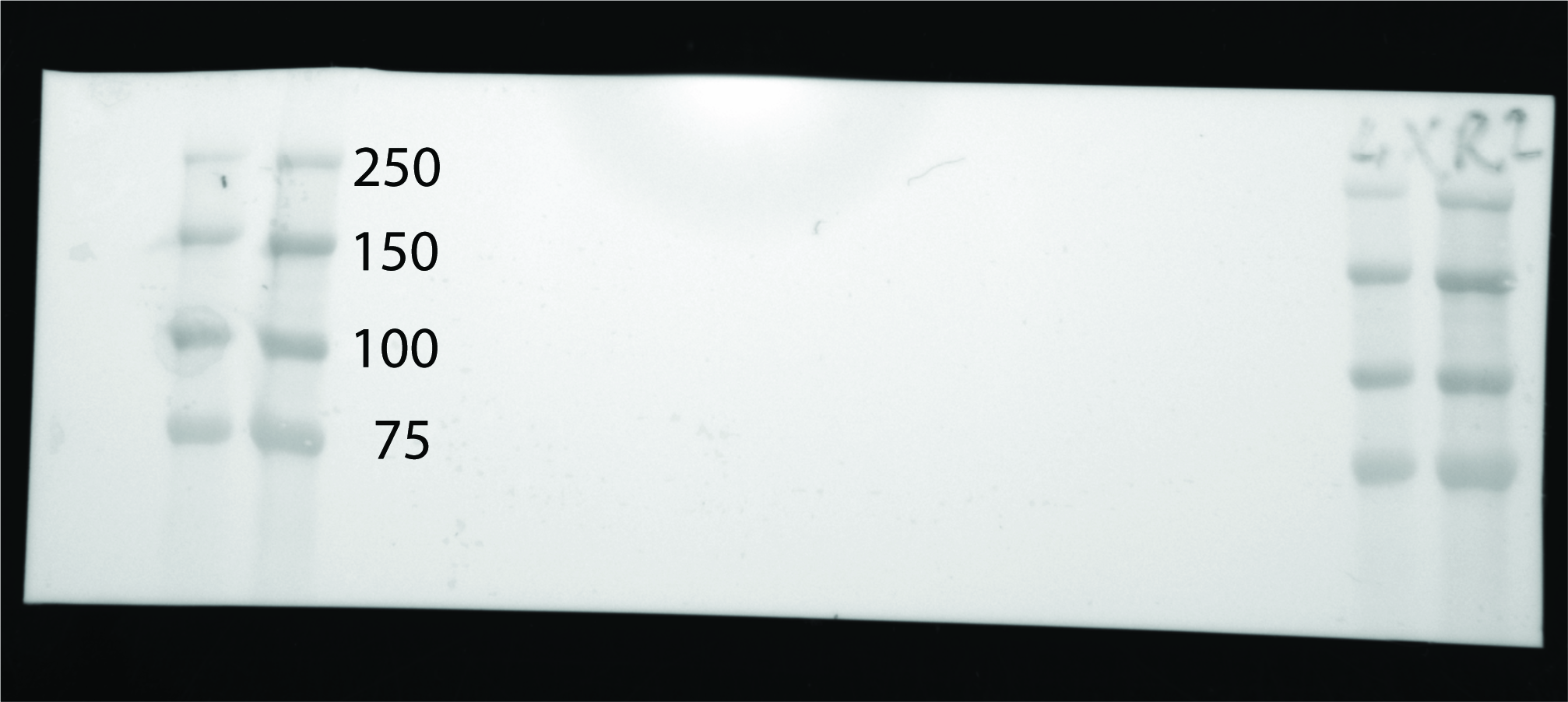

Supplement: Supplementary file 7 — Source data Fig. 6 [file 44320_2024_40_MOESM7_ESM.zip › Figure 6_Source Data/I/Image Data/colorimetric_TBK1_momelotinib_4X_r2.tif]

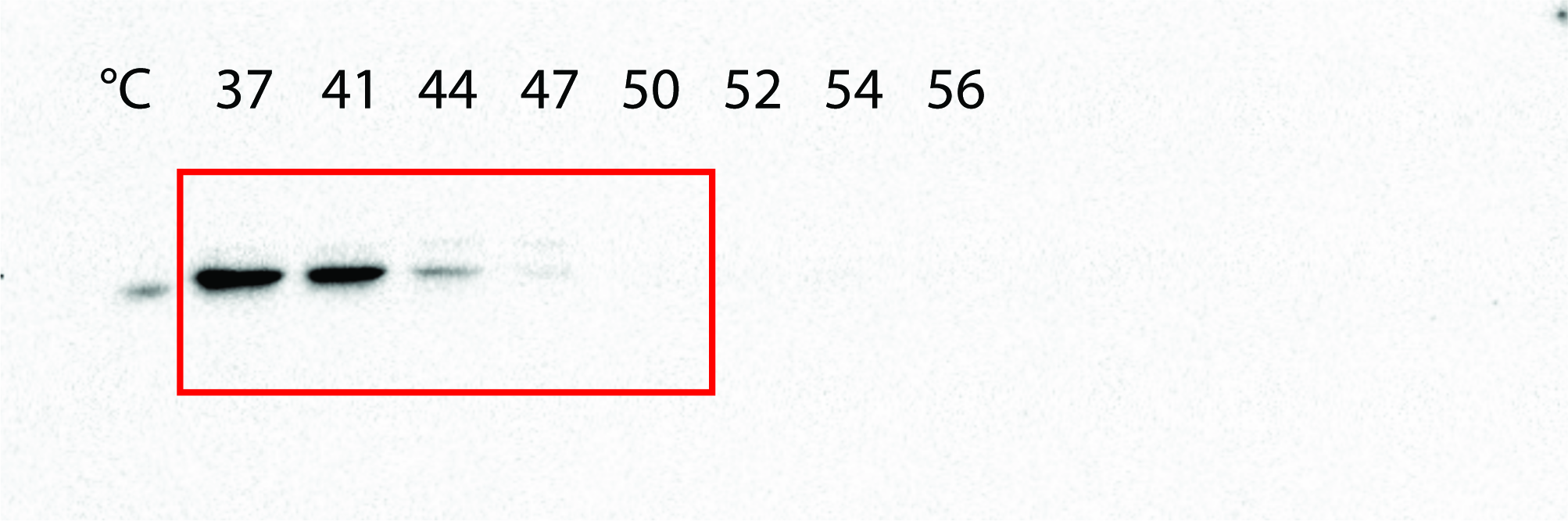

Supplement: Supplementary file 7 — Source data Fig. 6 [file 44320_2024_40_MOESM7_ESM.zip › Figure 6_Source Data/I/Image Data/western_TBK1_momelotinib_1X_r1.tif]

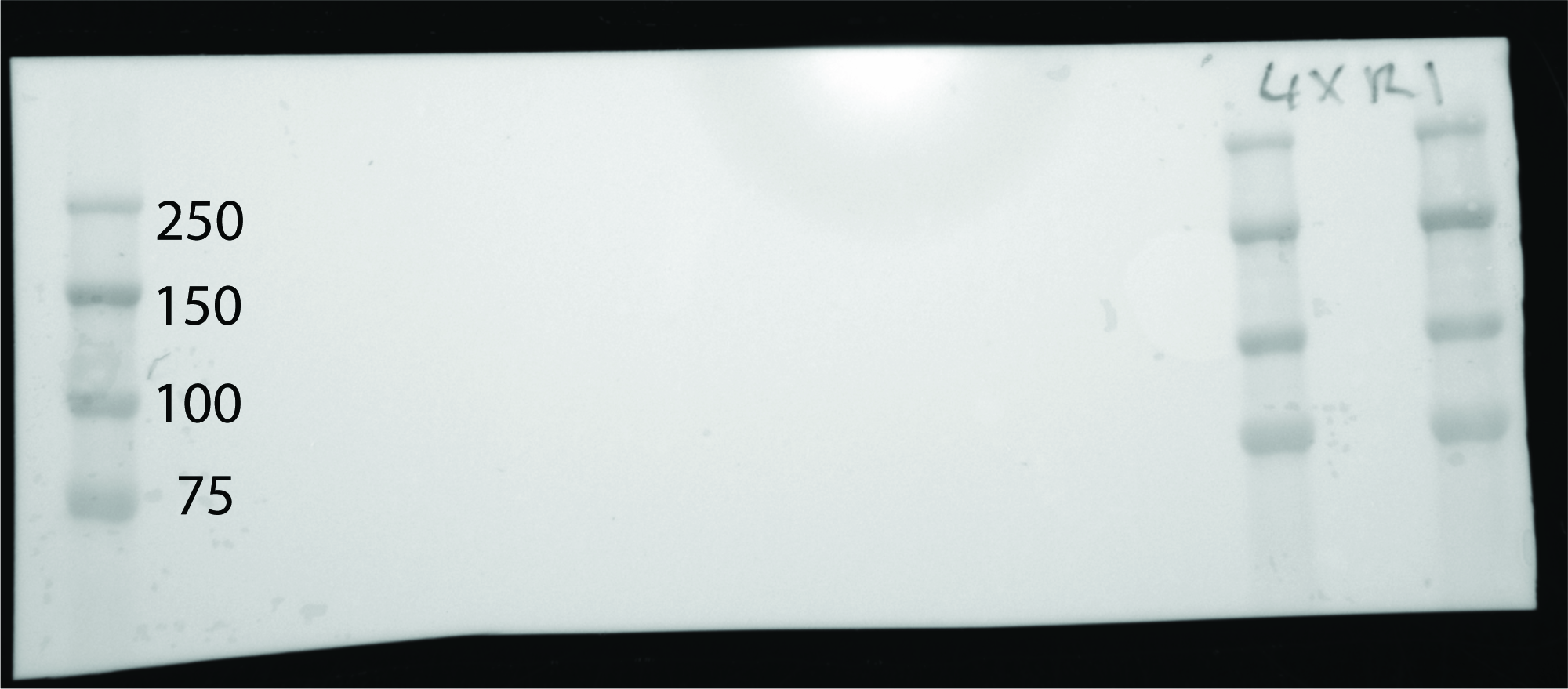

Supplement: Supplementary file 7 — Source data Fig. 6 [file 44320_2024_40_MOESM7_ESM.zip › Figure 6_Source Data/I/Image Data/colorimetric_TBK1_momelotinib_4X_r1.tif]

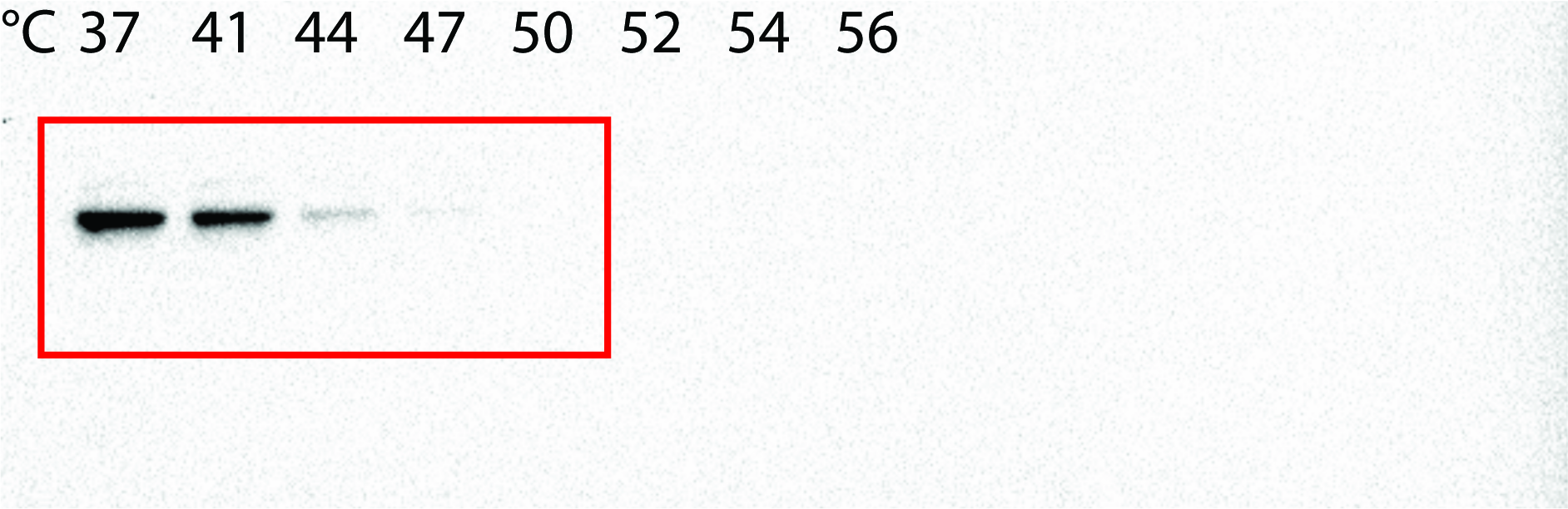

Supplement: Supplementary file 7 — Source data Fig. 6 [file 44320_2024_40_MOESM7_ESM.zip › Figure 6_Source Data/I/Image Data/western_TBK1_momelotinib_1X_r2.tif]

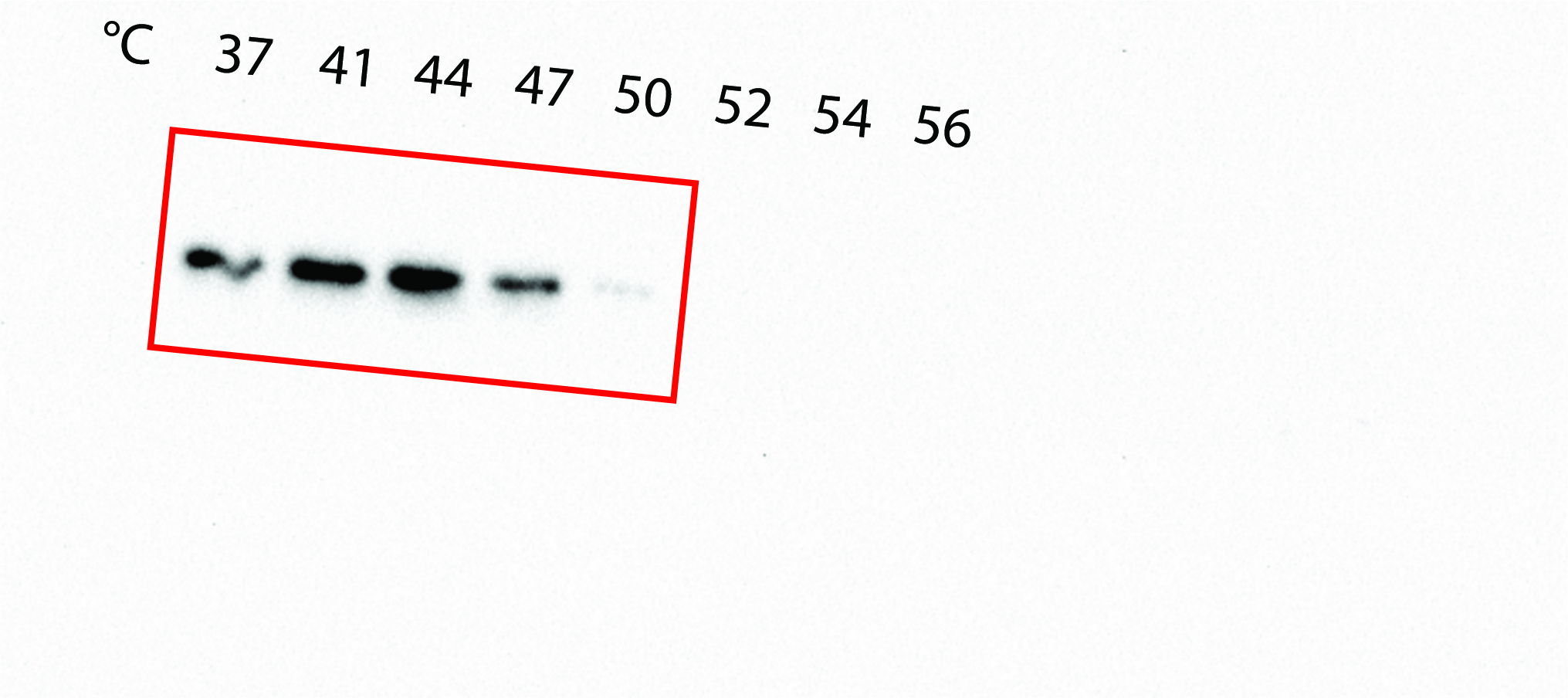

Supplement: Supplementary file 7 — Source data Fig. 6 [file 44320_2024_40_MOESM7_ESM.zip › Figure 6_Source Data/I/Image Data/western_GAPDH_momelotinib_4X_r1.tif]

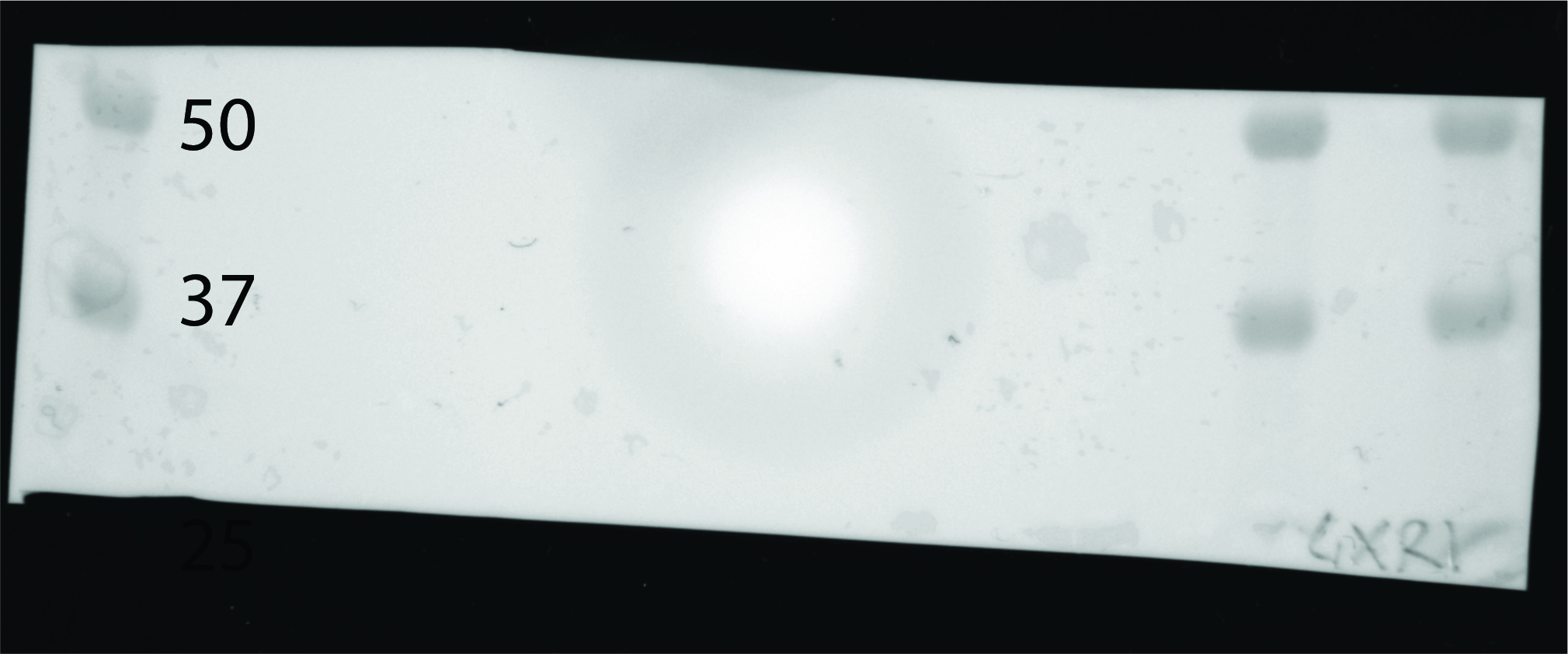

Supplement: Supplementary file 7 — Source data Fig. 6 [file 44320_2024_40_MOESM7_ESM.zip › Figure 6_Source Data/I/Image Data/colorimetric_GAPDH_momelotinib_4X_r1.tif]

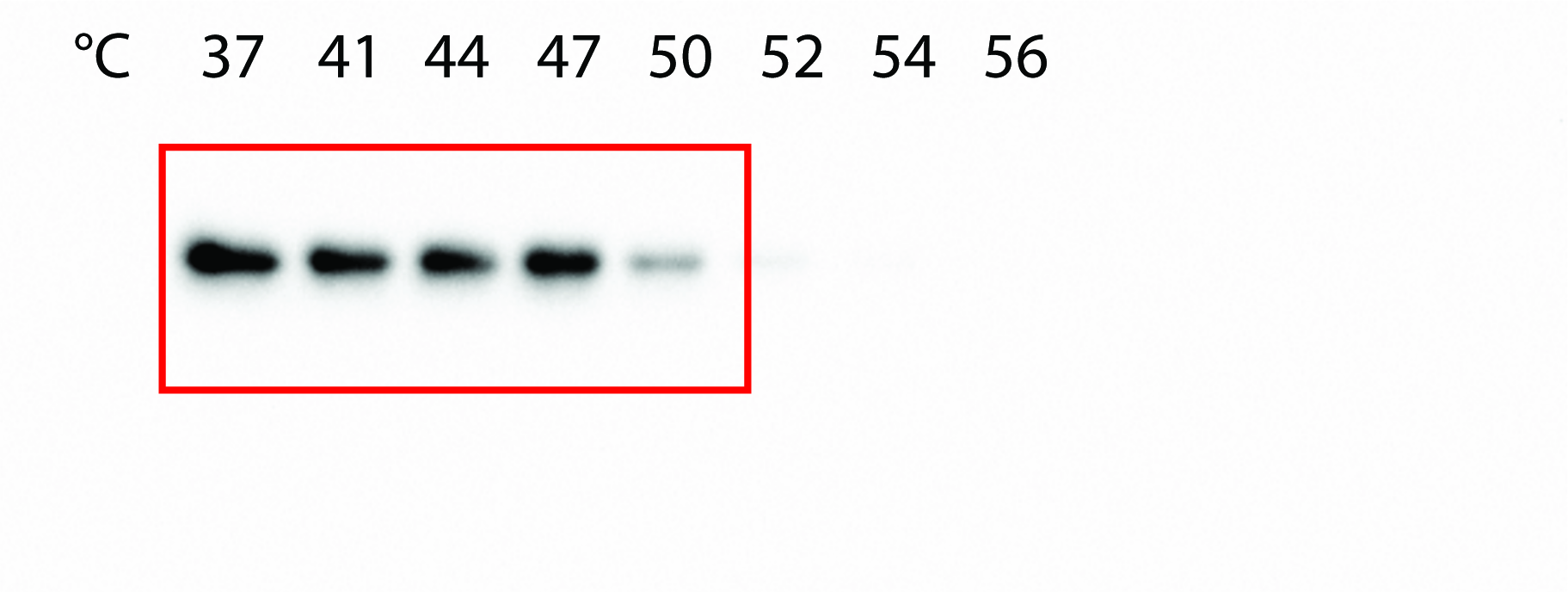

Supplement: Supplementary file 7 — Source data Fig. 6 [file 44320_2024_40_MOESM7_ESM.zip › Figure 6_Source Data/I/Image Data/western_GAPDH_momelotinib_4X_r2.tif]

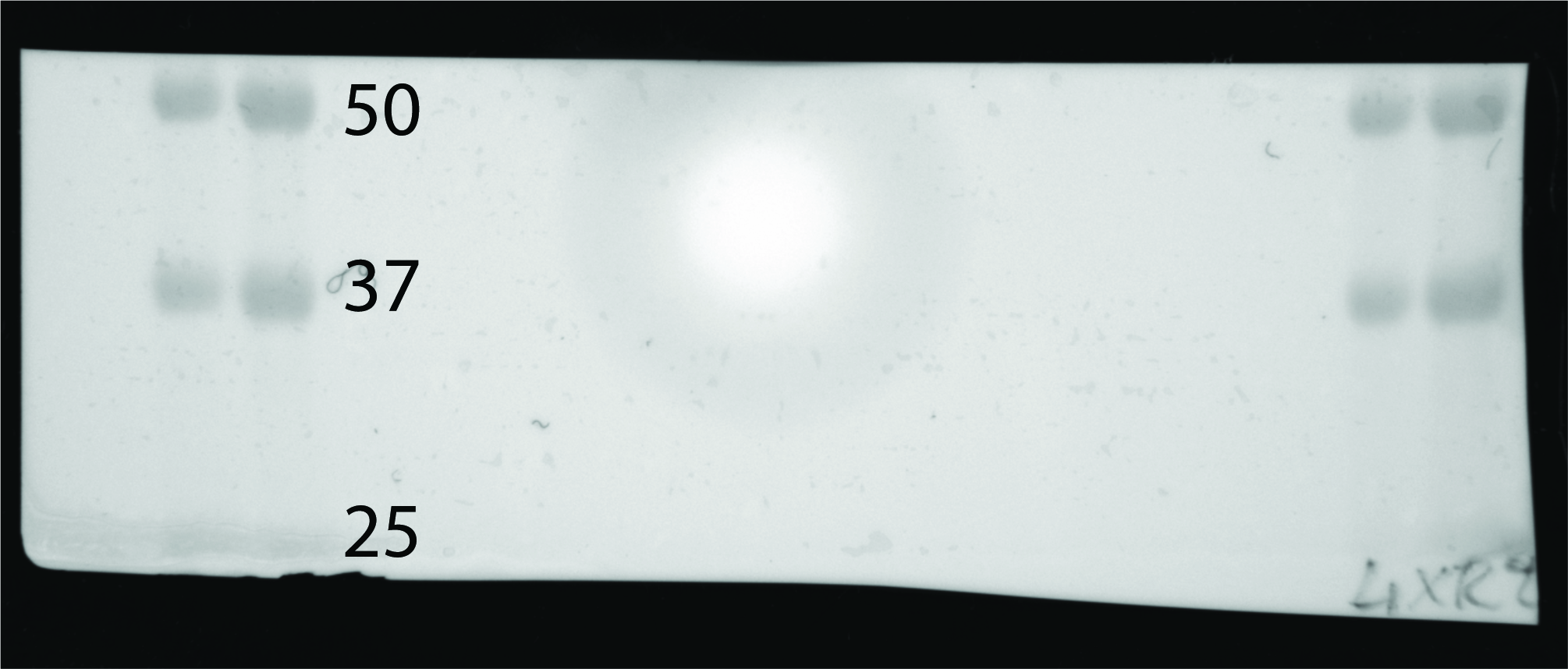

Supplement: Supplementary file 7 — Source data Fig. 6 [file 44320_2024_40_MOESM7_ESM.zip › Figure 6_Source Data/I/Image Data/colorimetric_GAPDH_momelotinib_4X_r2.tif]

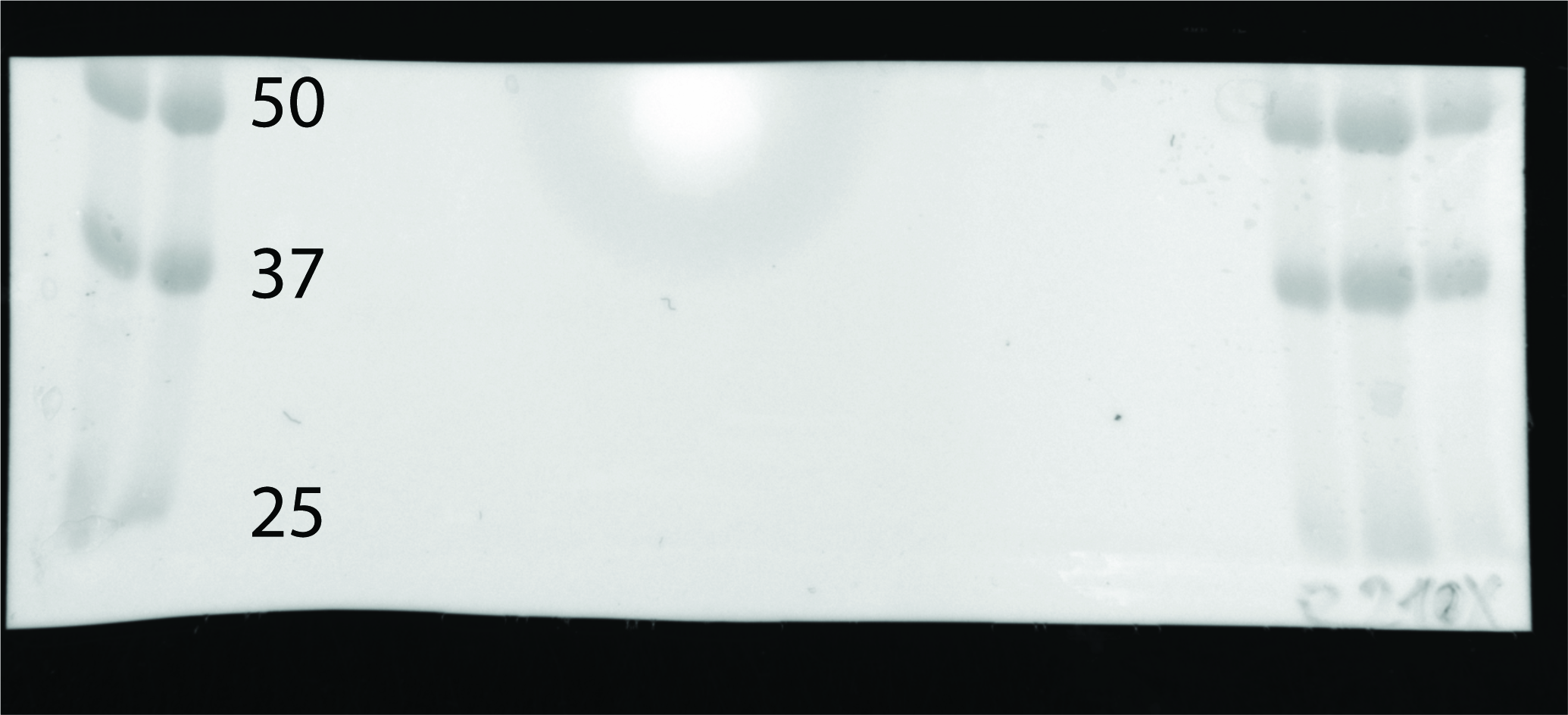

Supplement: Supplementary file 7 — Source data Fig. 6 [file 44320_2024_40_MOESM7_ESM.zip › Figure 6_Source Data/I/Image Data/colorimetric_GAPDH_momelotinib_10X_r2.tif]

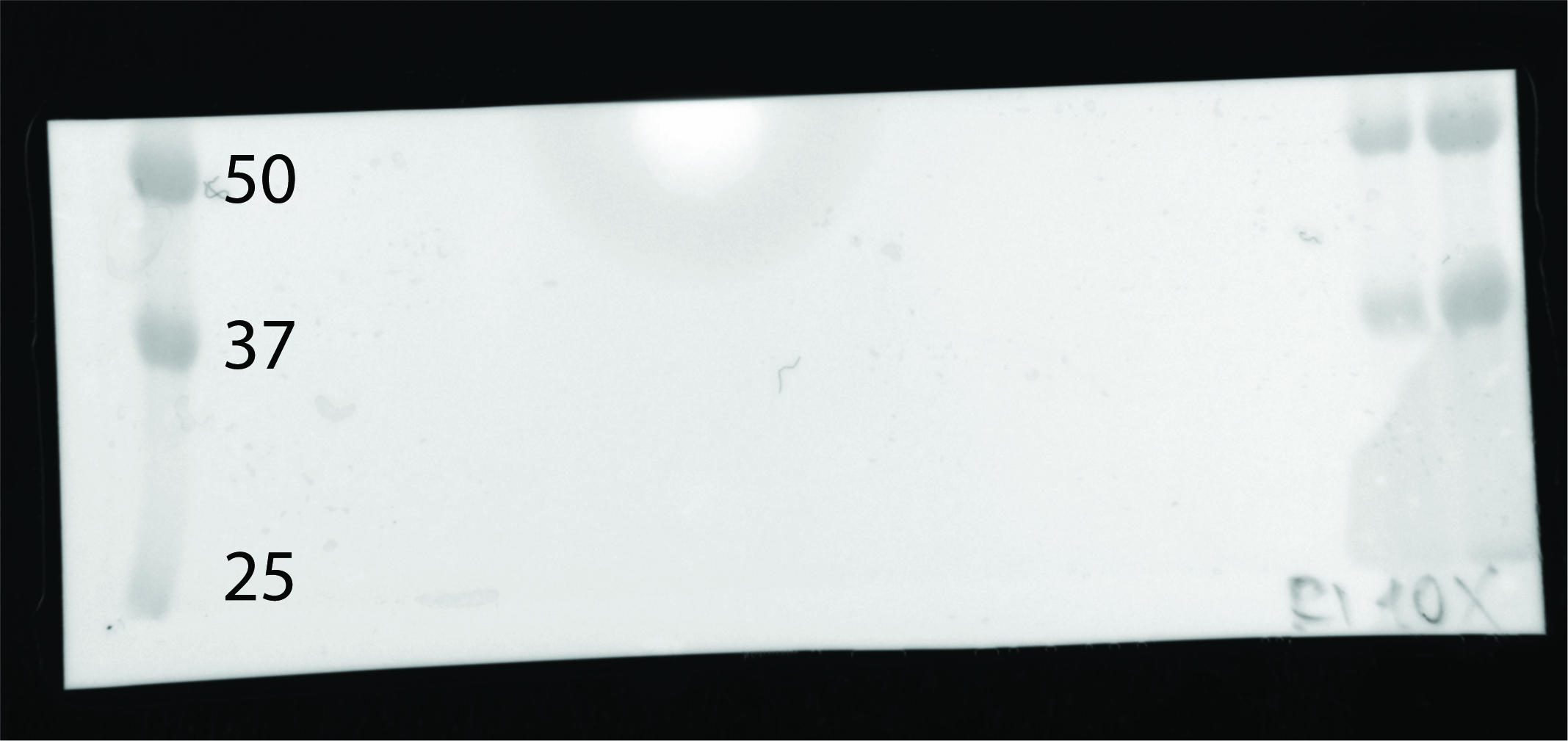

Supplement: Supplementary file 7 — Source data Fig. 6 [file 44320_2024_40_MOESM7_ESM.zip › Figure 6_Source Data/I/Image Data/colorimetric_GAPDH_momelotinib_10X_r1.tif]

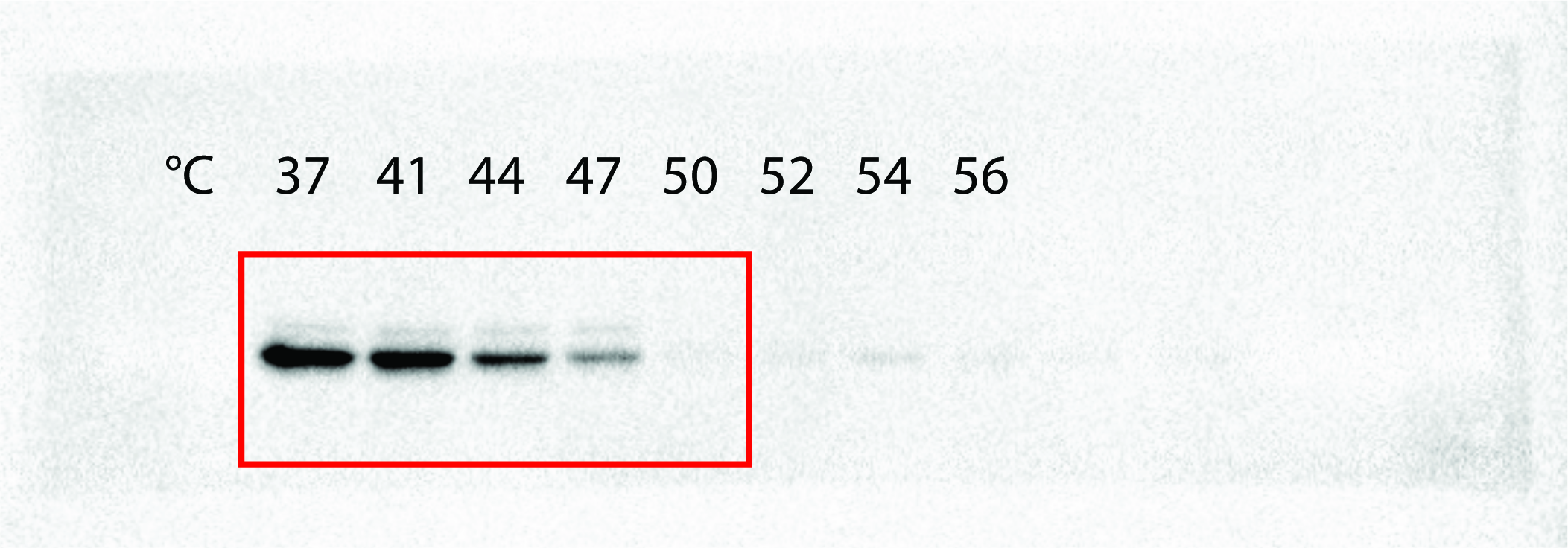

Supplement: Supplementary file 7 — Source data Fig. 6 [file 44320_2024_40_MOESM7_ESM.zip › Figure 6_Source Data/I/Image Data/western_TBK1_momelotinib_10X_r2.tif]

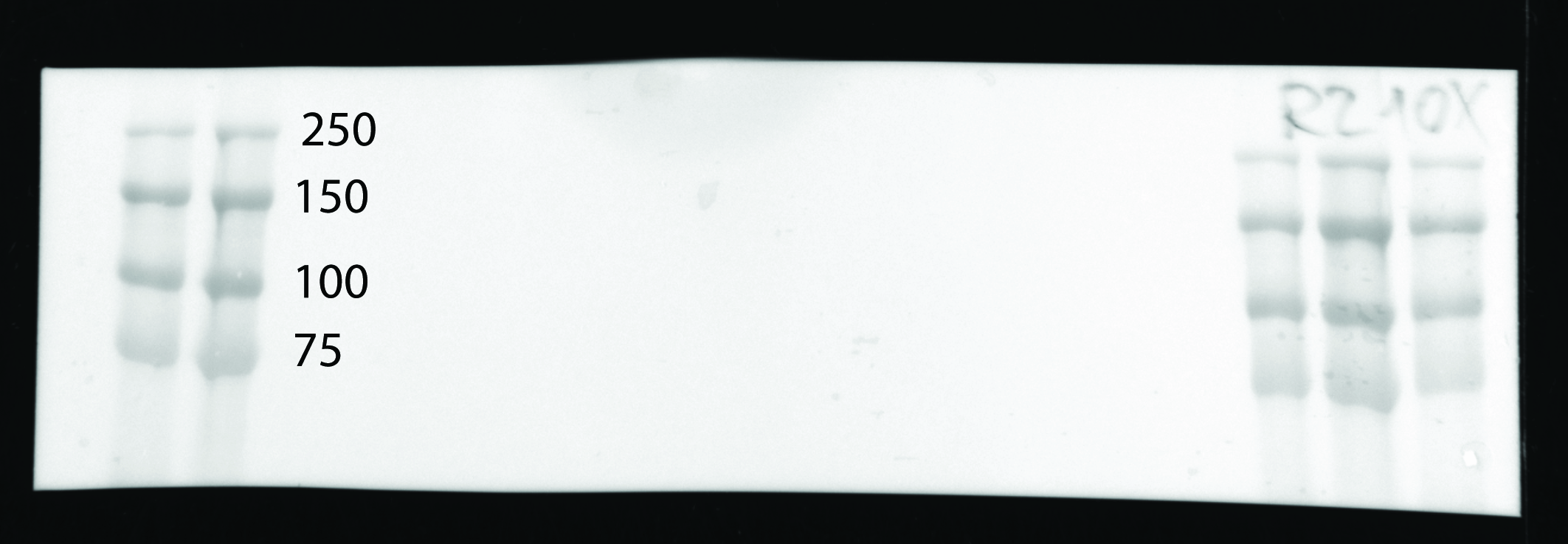

Supplement: Supplementary file 7 — Source data Fig. 6 [file 44320_2024_40_MOESM7_ESM.zip › Figure 6_Source Data/I/Image Data/colorimetric_TBK1_momelotinib_10X_r2.tif]

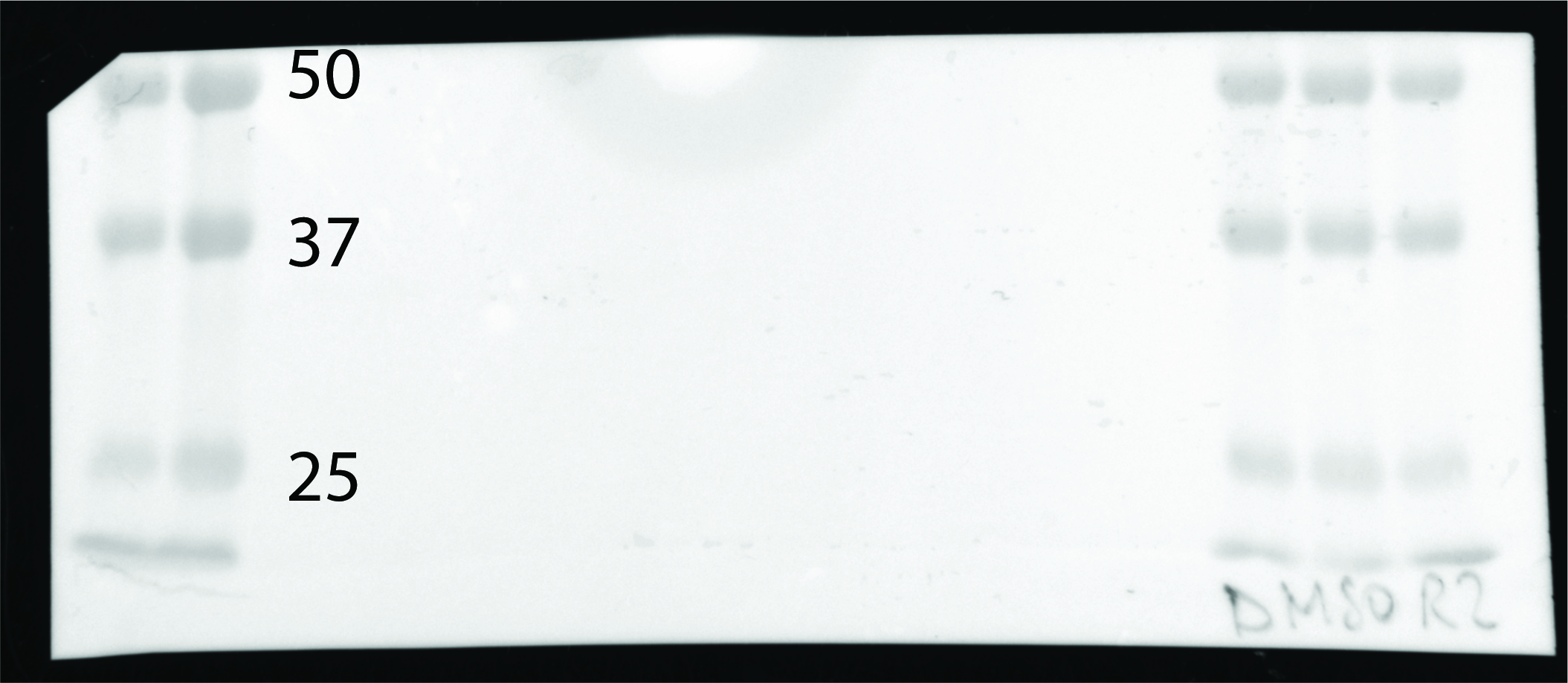

Supplement: Supplementary file 7 — Source data Fig. 6 [file 44320_2024_40_MOESM7_ESM.zip › Figure 6_Source Data/I/Image Data/colorimetric_GAPDH_dmso_r2.tif]

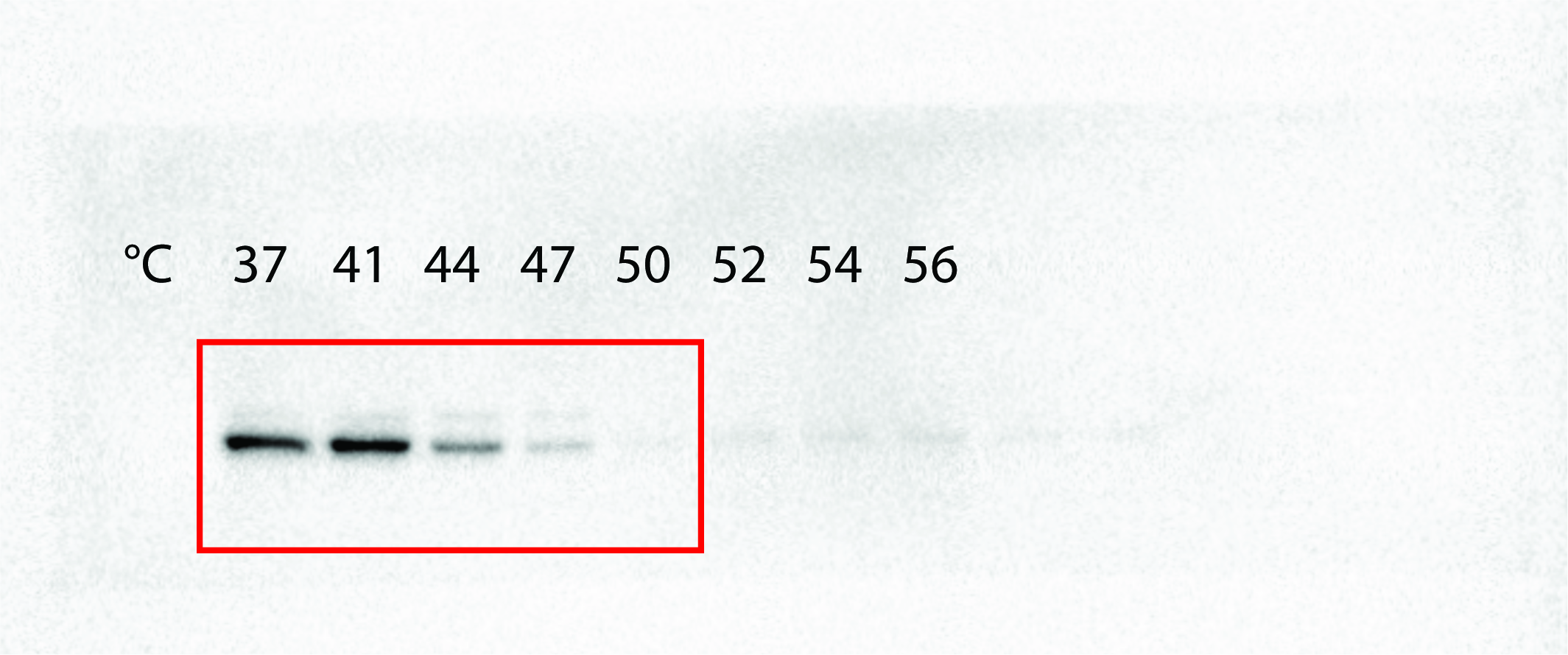

Supplement: Supplementary file 7 — Source data Fig. 6 [file 44320_2024_40_MOESM7_ESM.zip › Figure 6_Source Data/I/Image Data/western_TBK1_momelotinib_10X_r1.tif]

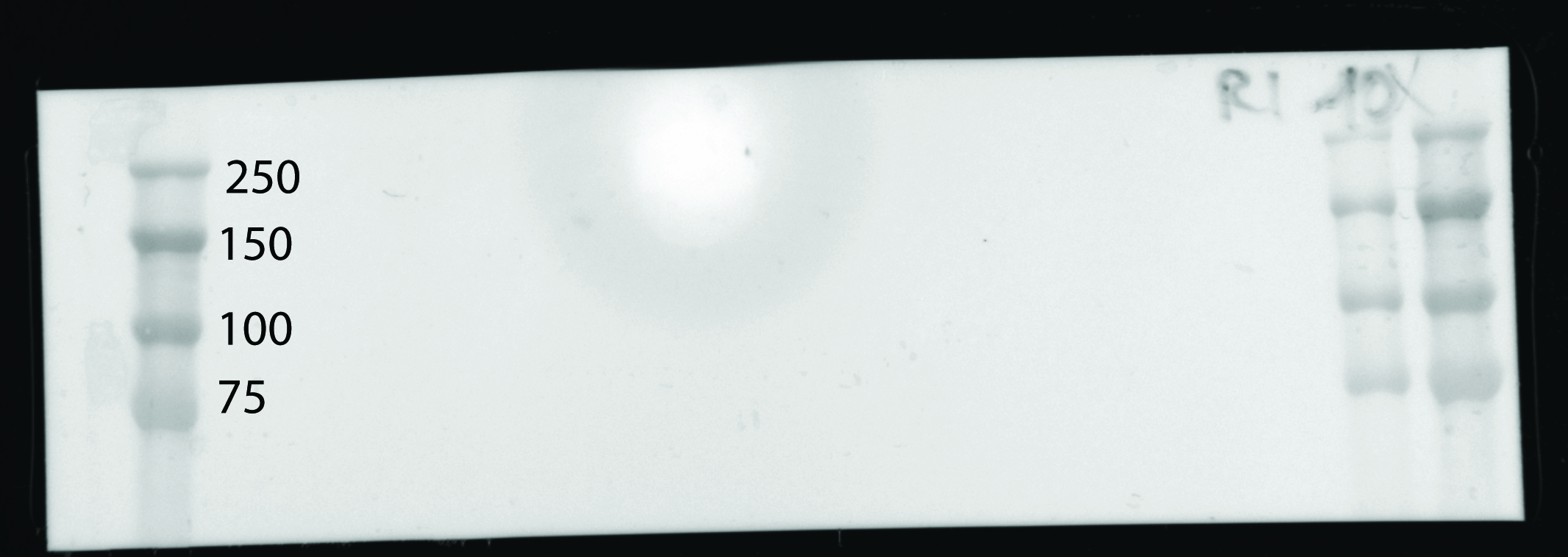

Supplement: Supplementary file 7 — Source data Fig. 6 [file 44320_2024_40_MOESM7_ESM.zip › Figure 6_Source Data/I/Image Data/colorimetric_TBK1_momelotinib_10X_r1.tif]

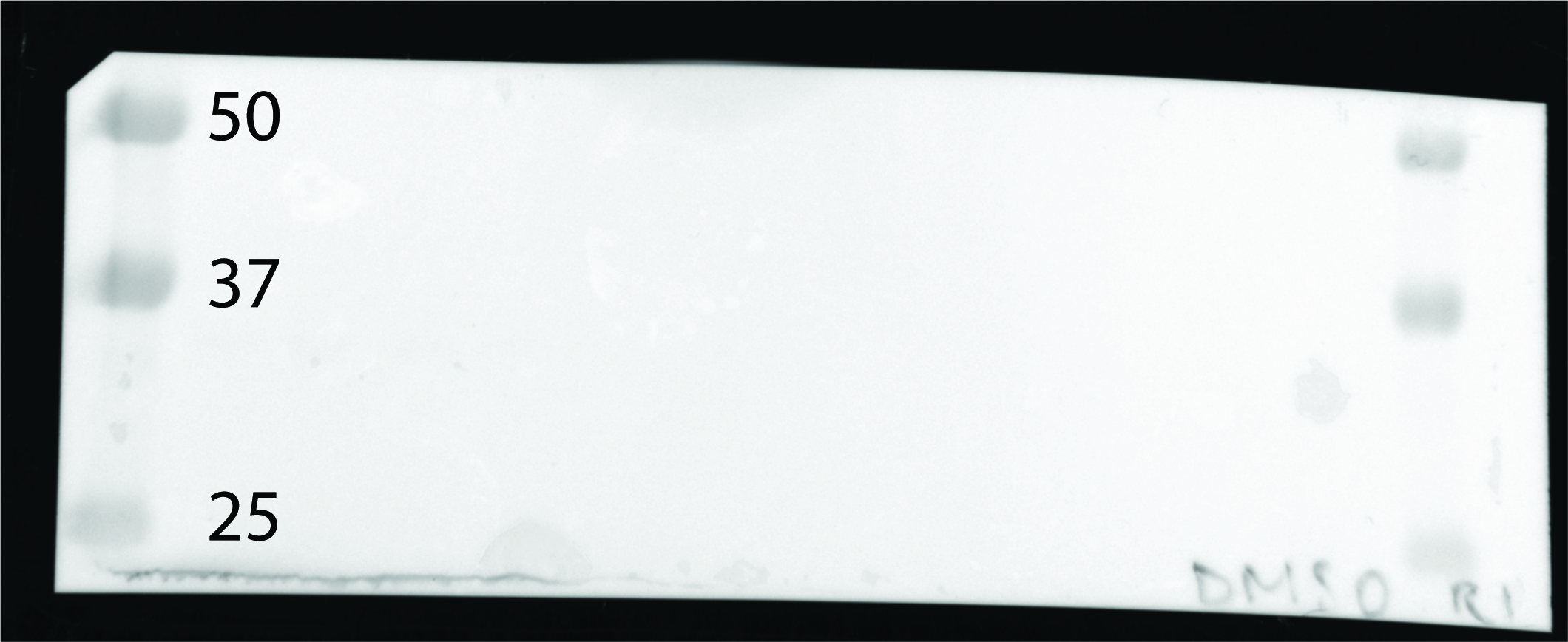

Supplement: Supplementary file 7 — Source data Fig. 6 [file 44320_2024_40_MOESM7_ESM.zip › Figure 6_Source Data/I/Image Data/colorimetric_GAPDH_dmso_r1.tif]

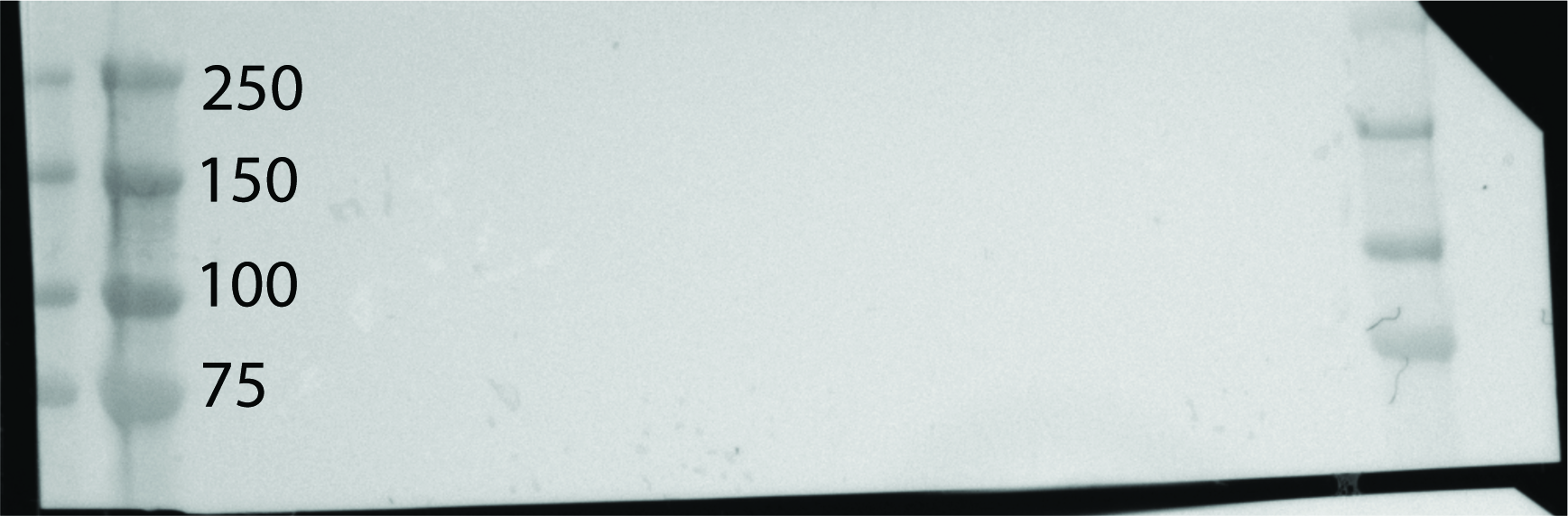

Supplement: Supplementary file 7 — Source data Fig. 6 [file 44320_2024_40_MOESM7_ESM.zip › Figure 6_Source Data/H/Image Data/colorimetric_TBK1_dmso_r1.tif]

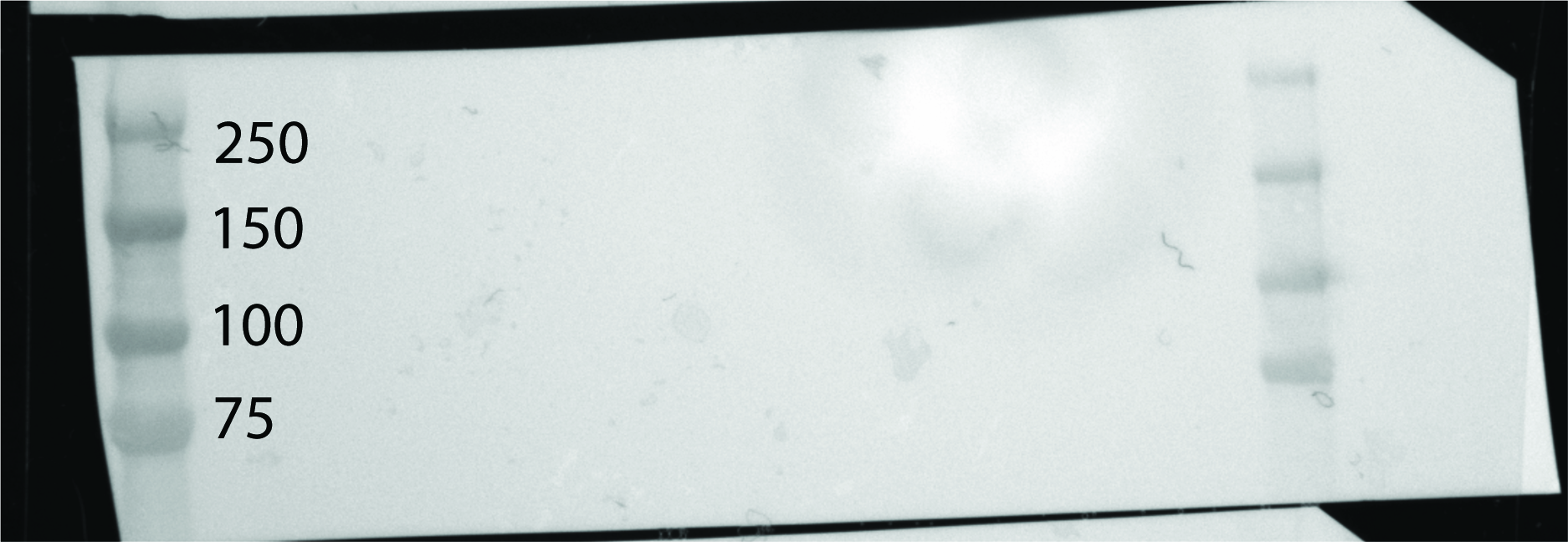

Supplement: Supplementary file 7 — Source data Fig. 6 [file 44320_2024_40_MOESM7_ESM.zip › Figure 6_Source Data/H/Image Data/colorimetric_TBK1_midostaurin_r1.tif]

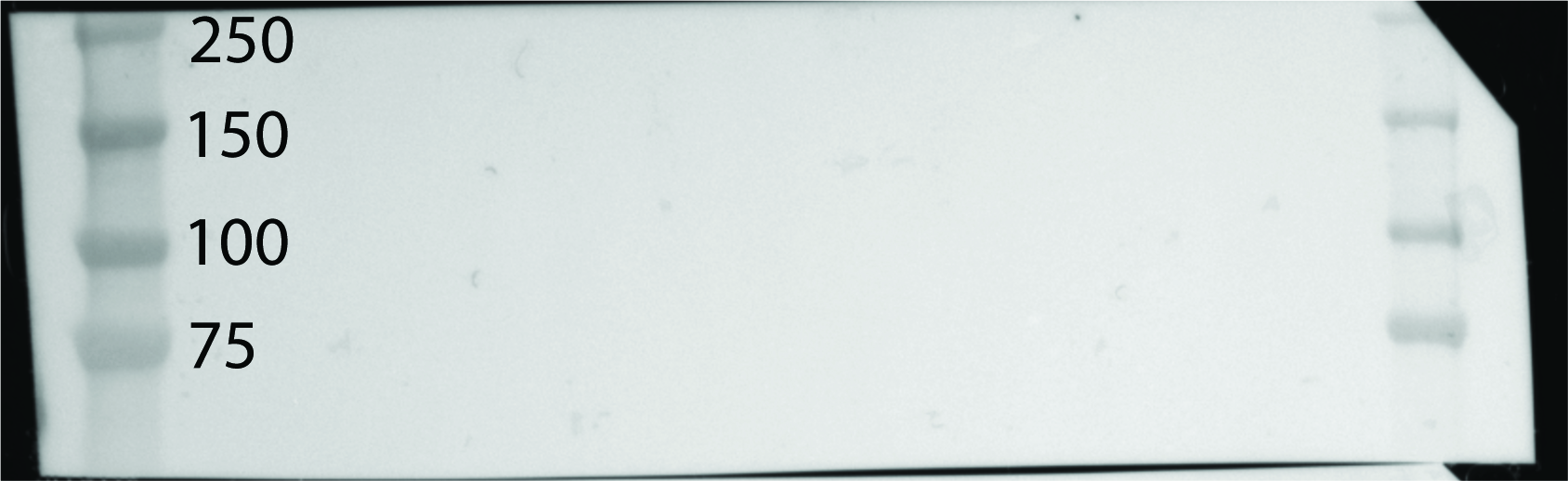

Supplement: Supplementary file 7 — Source data Fig. 6 [file 44320_2024_40_MOESM7_ESM.zip › Figure 6_Source Data/H/Image Data/colorimetric_TBK1_dmso_r2.tif]

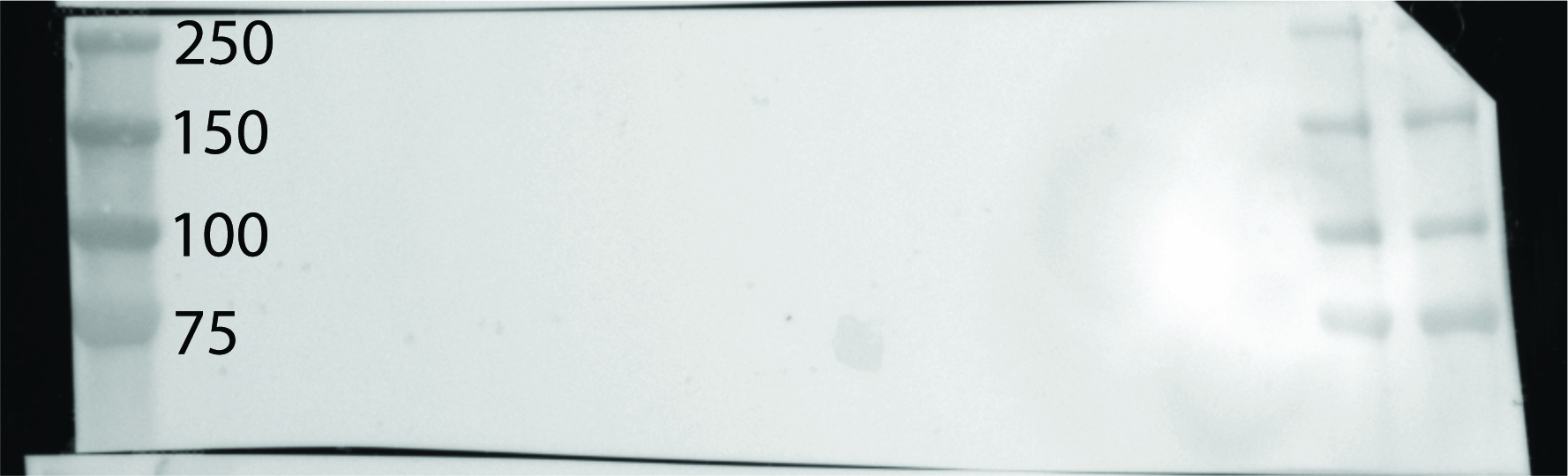

Supplement: Supplementary file 7 — Source data Fig. 6 [file 44320_2024_40_MOESM7_ESM.zip › Figure 6_Source Data/H/Image Data/colorimetric_TBK1_midostaurin_r2.tif]

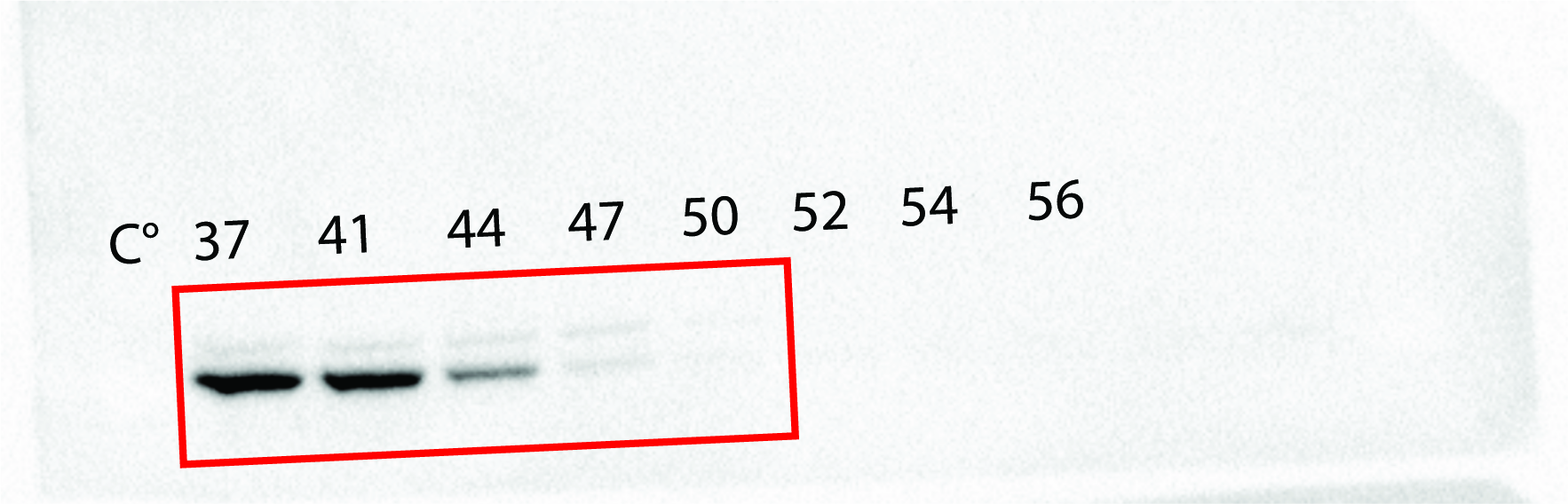

Supplement: Supplementary file 7 — Source data Fig. 6 [file 44320_2024_40_MOESM7_ESM.zip › Figure 6_Source Data/H/Image Data/western_TBK1_dmso_r1.tif]

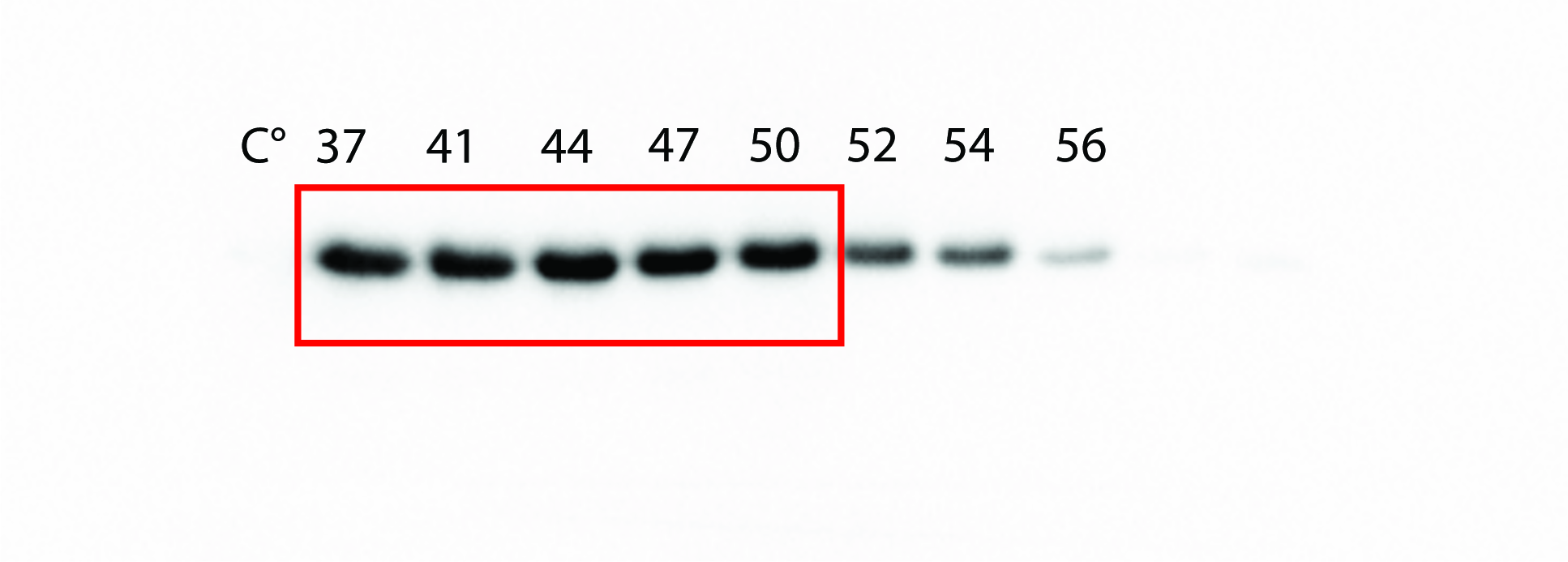

Supplement: Supplementary file 7 — Source data Fig. 6 [file 44320_2024_40_MOESM7_ESM.zip › Figure 6_Source Data/H/Image Data/western_GAPDH_dmso_r2.tif]

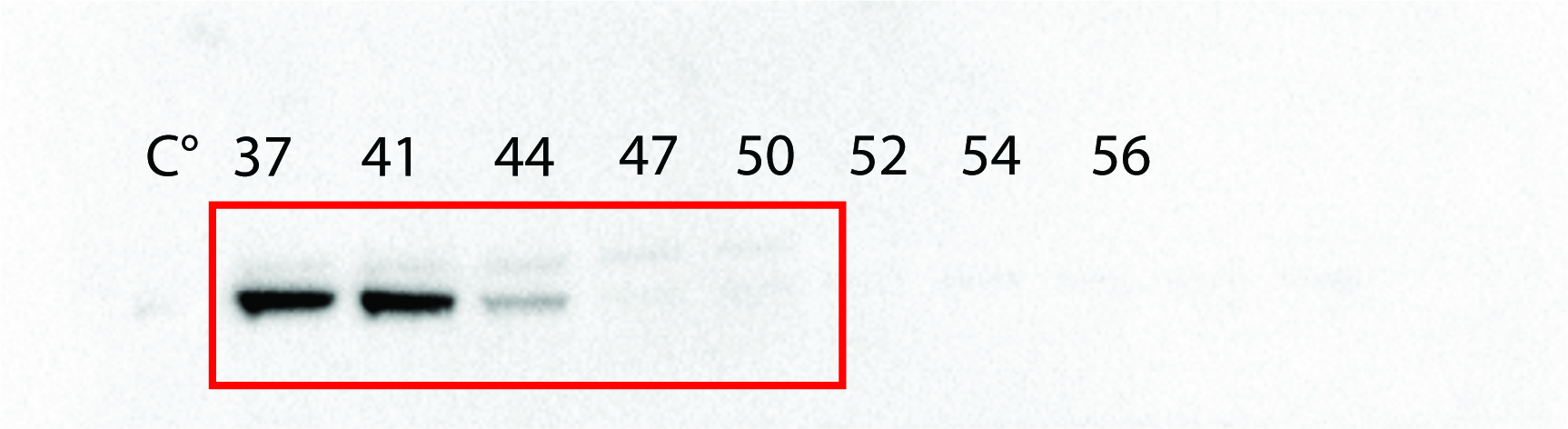

Supplement: Supplementary file 7 — Source data Fig. 6 [file 44320_2024_40_MOESM7_ESM.zip › Figure 6_Source Data/H/Image Data/western_TBK1_dmso_r2.tif]

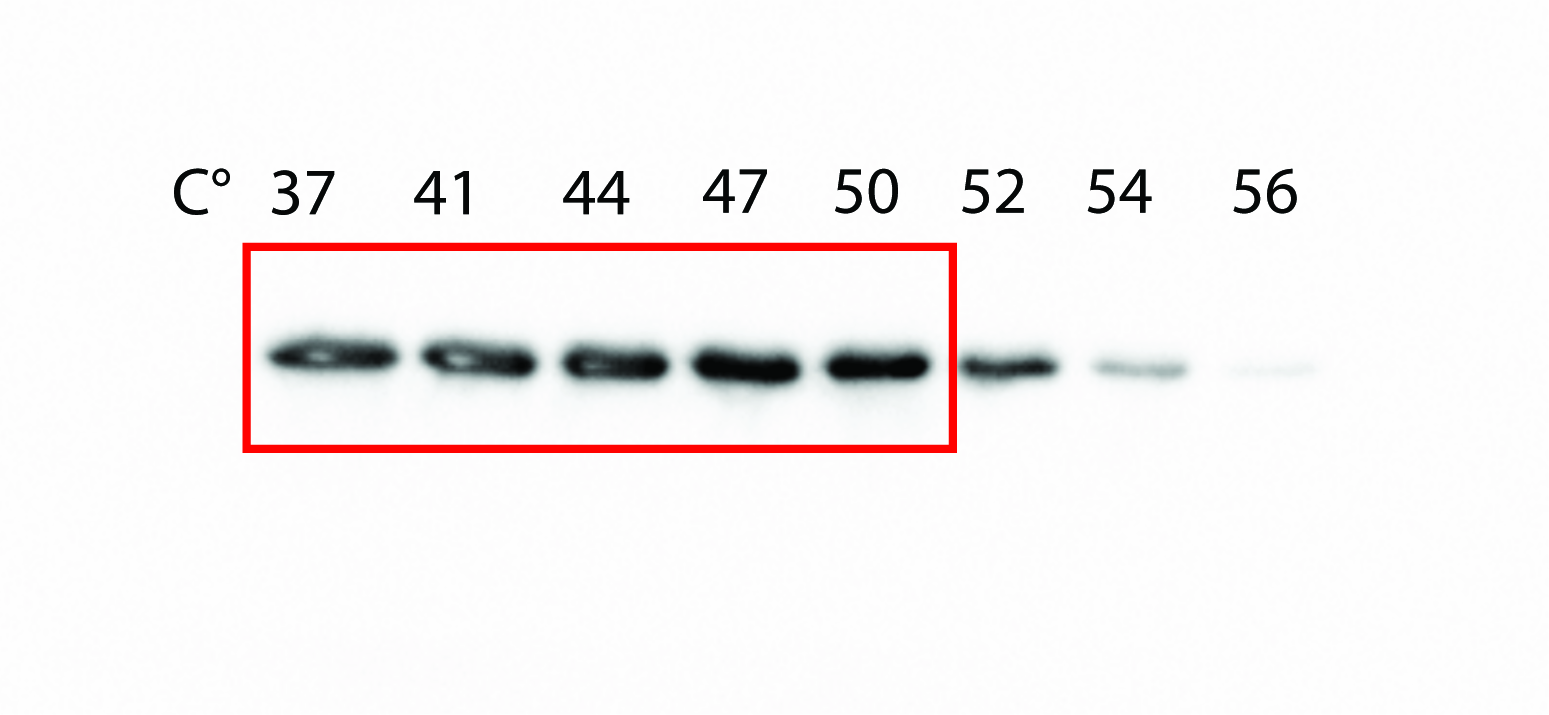

Supplement: Supplementary file 7 — Source data Fig. 6 [file 44320_2024_40_MOESM7_ESM.zip › Figure 6_Source Data/H/Image Data/western_GAPDH_dmso_r1.tif]

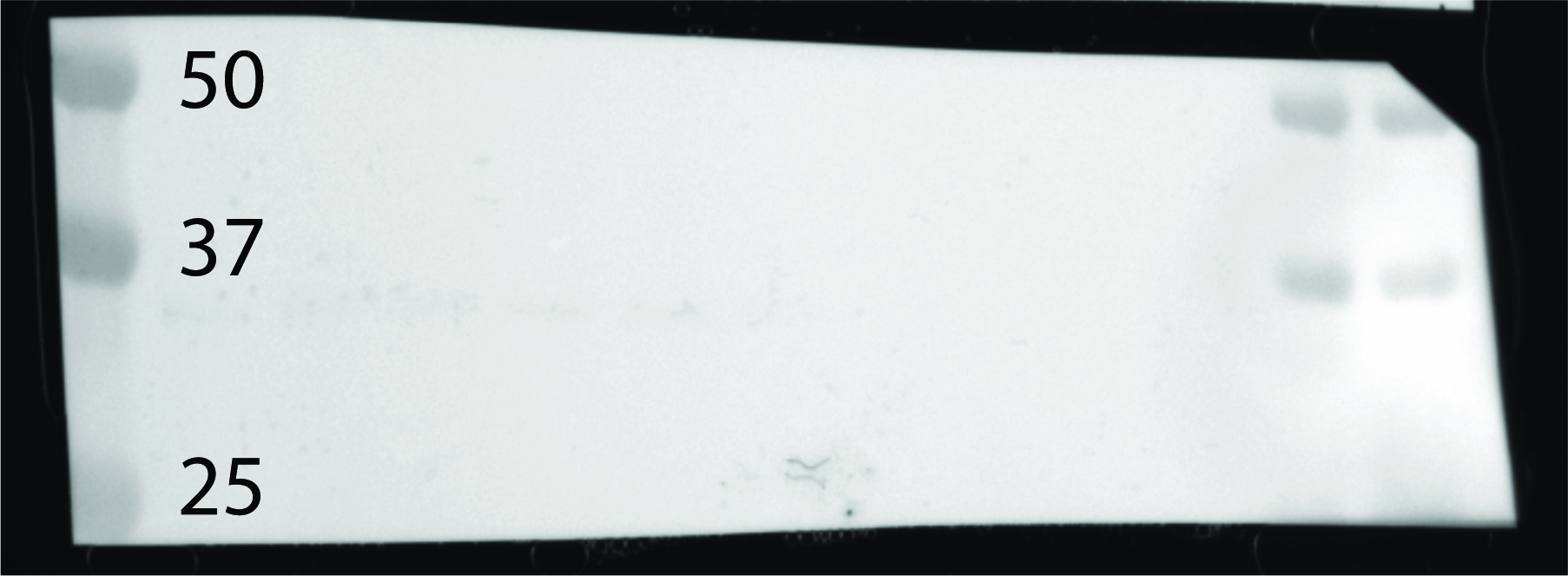

Supplement: Supplementary file 7 — Source data Fig. 6 [file 44320_2024_40_MOESM7_ESM.zip › Figure 6_Source Data/H/Image Data/colorimetric_GAPDH_midostaurin_r2.tif]

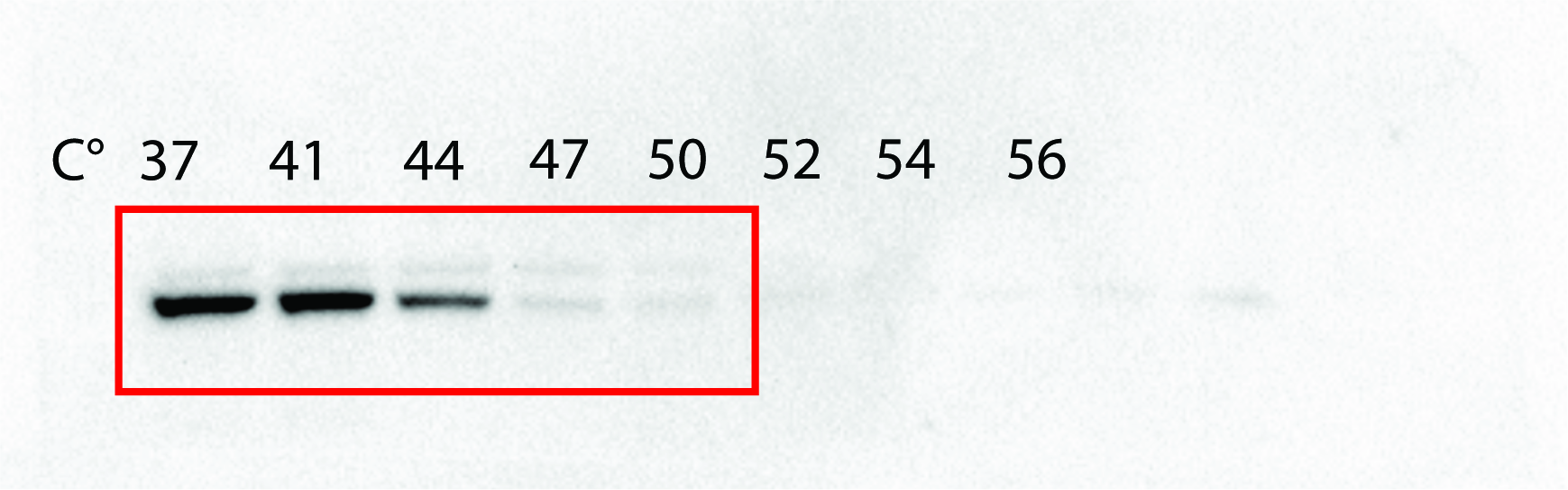

Supplement: Supplementary file 7 — Source data Fig. 6 [file 44320_2024_40_MOESM7_ESM.zip › Figure 6_Source Data/H/Image Data/western_TBK1_midostaurin_r2.tif]

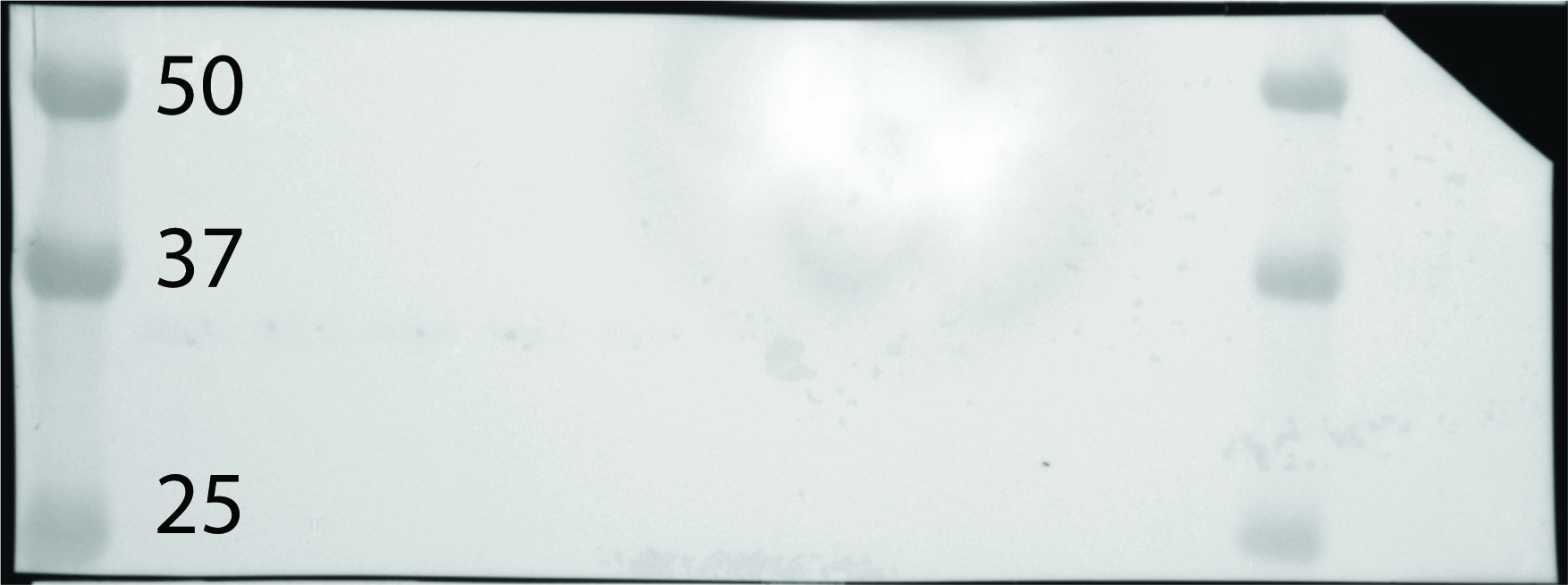

Supplement: Supplementary file 7 — Source data Fig. 6 [file 44320_2024_40_MOESM7_ESM.zip › Figure 6_Source Data/H/Image Data/colorimetric_GAPDH_midostaurin_r1.tif]

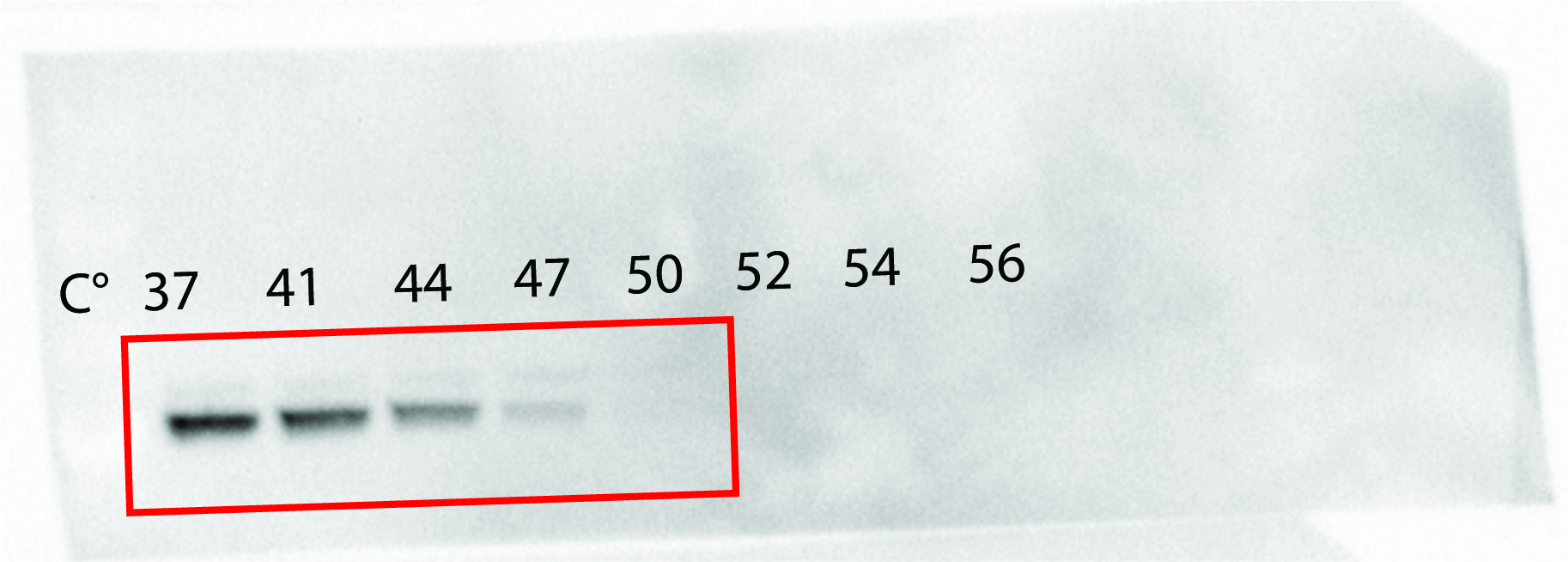

Supplement: Supplementary file 7 — Source data Fig. 6 [file 44320_2024_40_MOESM7_ESM.zip › Figure 6_Source Data/H/Image Data/western_TBK1_midostaurin_r1.tif]

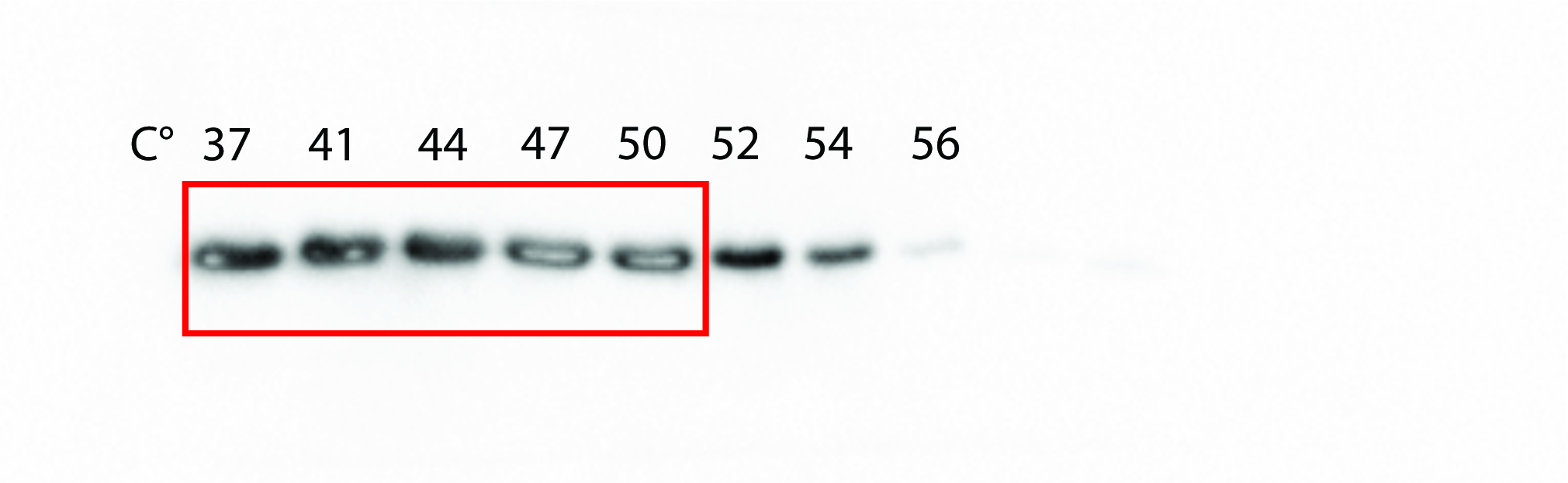

Supplement: Supplementary file 7 — Source data Fig. 6 [file 44320_2024_40_MOESM7_ESM.zip › Figure 6_Source Data/H/Image Data/western_GAPDH_midostaurin_r2.tif]

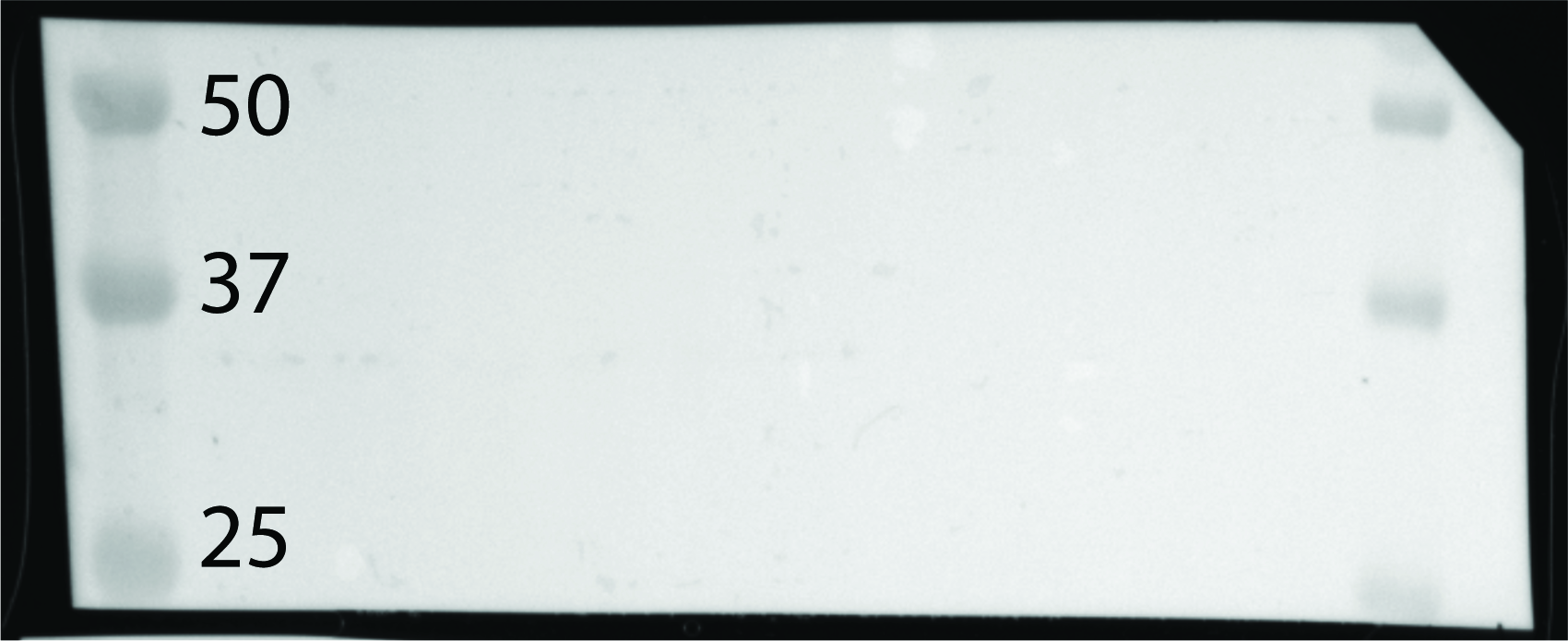

Supplement: Supplementary file 7 — Source data Fig. 6 [file 44320_2024_40_MOESM7_ESM.zip › Figure 6_Source Data/H/Image Data/colorimetric_GAPDH_dmso_r2.tif]

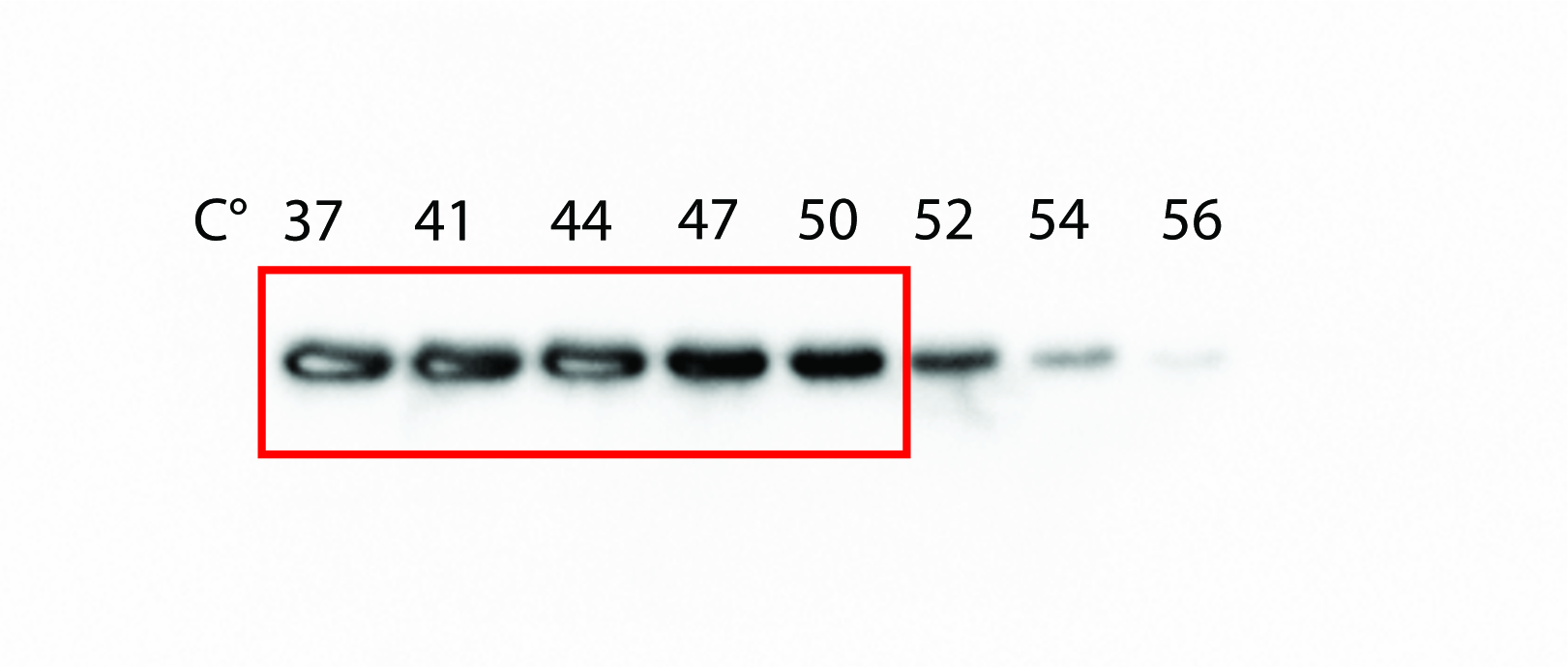

Supplement: Supplementary file 7 — Source data Fig. 6 [file 44320_2024_40_MOESM7_ESM.zip › Figure 6_Source Data/H/Image Data/western_GAPDH_midostaurin_r1.tif]

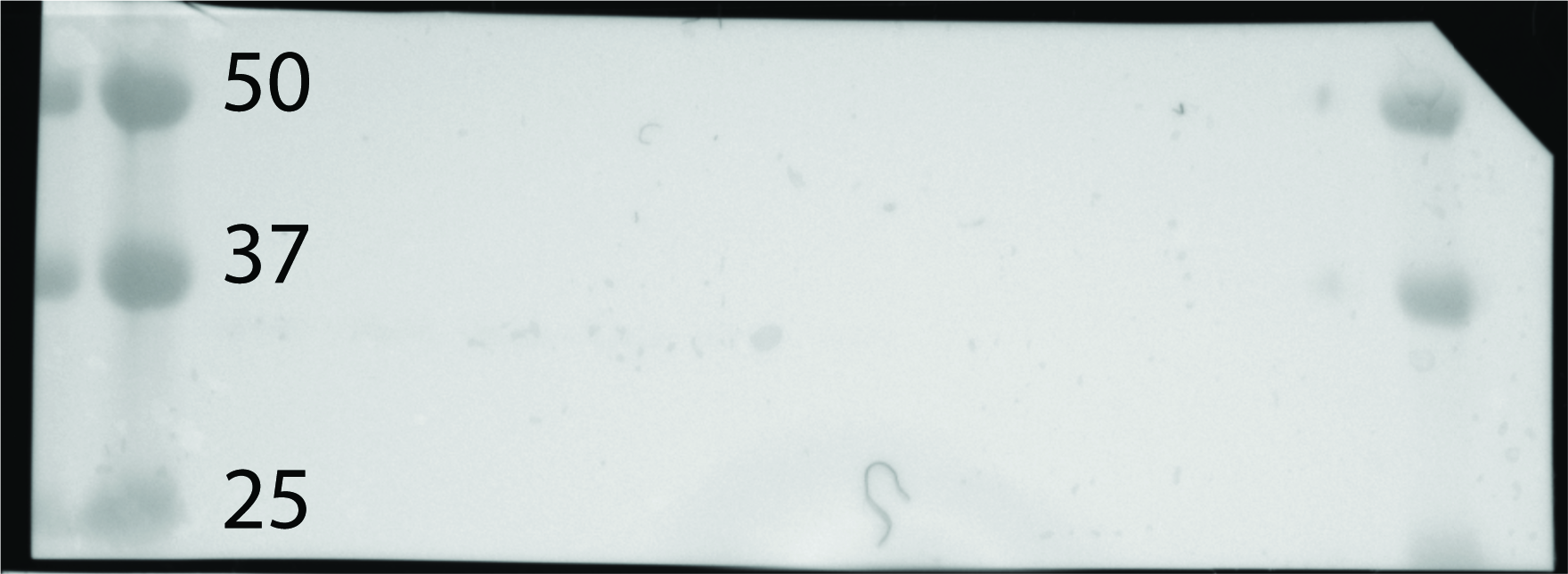

Supplement: Supplementary file 7 — Source data Fig. 6 [file 44320_2024_40_MOESM7_ESM.zip › Figure 6_Source Data/H/Image Data/colorimetric_GAPDH_dmso_r1.tif]
